# Diversity and Epidemiology of Bat Trypanosomes: A One Health Perspective

**DOI:** 10.3390/pathogens10091148

**Published:** 2021-09-06

**Authors:** Jill M. Austen, Amanda D. Barbosa

**Affiliations:** 1 Centre for Biosecurity and One Health, Harry Butler Institute, Murdoch University, Murdoch, WA 6150, Australia; 2 CAPES Foundation, Ministry of Education of Brazil, Brasilia 70040-020, DF, Brazil

**Keywords:** bat, *Trypanosoma*, diversity, molecular epidemiology, transmission cycle, zoonosis, One Health

## Abstract

Bats (order Chiroptera) have been increasingly recognised as important reservoir hosts for human and animal pathogens worldwide. In this context, molecular and microscopy-based investigations to date have revealed remarkably high diversity of *Trypanosoma* spp. harboured by bats, including species of recognised medical and veterinary importance such as *Trypanosoma cruzi* and *Trypanosoma evansi* (aetiological agents of Chagas disease and Surra, respectively). This review synthesises current knowledge on the diversity, taxonomy, evolution and epidemiology of bat trypanosomes based on both molecular studies and morphological records. In addition, we use a One Health approach to discuss the significance of bats as reservoirs (and putative vectors) of *T. cruzi*, with a focus on the complex associations between intra-specific genetic diversity and eco-epidemiology of *T. cruzi* in sylvatic and domestic ecosystems. This article also highlights current knowledge gaps on the biological implications of trypanosome co-infections in a single host, as well as the prevalence, vectors, life-cycle, host-range and clinical impact of most bat trypanosomes recorded to date. Continuous research efforts involving molecular surveillance of bat trypanosomes are required for improved disease prevention and control, mitigation of biosecurity risks and potential spill-over events, ultimately ensuring the health of humans, domestic animals and wildlife globally.

## 1. Introduction

One Health is an approach that recognizes that the health of people is connected to the health of animals and their shared environment. This concept has become more important in recent years due to climate change, the emergence of infectious diseases that can quickly spread across borders and around the globe and the growth and expansion of human populations into new geographic areas, resulting in increased contact with wild and domestic animals [1].

A One Health approach to study the epidemiology, transmission and evolutionary dynamics of vector-borne diseases is crucial for the success of human and animal disease control programmes, particularly neglected diseases in low to middle income countries. These include, for example, dengue fever, leishmaniasis and Chagas disease (or American trypanosomiasis) which collectively represent a significant global health challenge [2,3,4].

The causative agents of Chagas disease (*Trypanosoma cruzi*) belong to the protist family Trypanosomatidae (Euglenozoa, Kinetoplastea). *Trypanosoma* spp. are ubiquitous flagellated protistan parasites of all classes of vertebrates, mainly transmitted via faecal or salivary infected material of blood-feeding arthropods and leeches [5]. Other transmission mechanisms such as vertical and oral transmission as well as through the bite of hematophagous bats are also possible [5,6,7,8].

In addition to impacting the health of people, trypanosomes are also known to cause important animal diseases such as surra (also known as murrina and ‘mal de caderas’ in South America), caused by *T. evansi*. This disease causes serious economic losses and is reported in cattle, horses, camels, buffaloes, deer, cats and endangered native mammals [6,9]. The geographic distribution of Surra comprises Northern Africa, Southeast Asia, Europe, and Central and South America [6,10].

Bats (order Chiroptera) are the second most diverse mammalian group after rodents, with 1390 recognized species across 227 genera [11,12]. These flying mammals have a widespread distribution and have been increasingly recognised as important reservoir hosts for pathogens that can cross species barriers to infect humans and other domestic and wild mammals [13]. For instance, bats have been implicated in the transmission of high-impact viral zoonoses such as severe acute respiratory syndrome coronaviruses (including COVID-19), Ebola, and Hendra and Nipah paramyxoviruses [14,15].

Bats are also known to harbour a high diversity of trypanosomes [5,16,17,18,19,20,21,22,23,24,25,26,27,28,29,30,31,32,33,34,35,36,37,38,39,40,41,42,43,44]. In addition to the well-known pathogens *T. cruzi* and *T. evansi,* another 48 trypanosome species and genotypes have been reported in more than 75 species of bats from a wide range of Chiroptera families in the American continent, Africa, Asia, Europe and Oceania (e.g., [6,26,45,46,47,48,49,50]). Unfortunately, genetic data are only available for 24 *Trypanosoma* spp., which were characterised after the advent and broader accessibility of molecular techniques.

Molecular and phylogenetic studies conducted to date have shown that the vast majority of bat trypanosomes described belong to the highly diverse *T. cruzi* clade. Importantly, these studies collectively have generated compelling evidence that bats played a role in the evolution of the zoonotic *T. cruzi* and its dispersion to a range of terrestrial mammal hosts, including humans [26,40,46,47,49,51].

Morphologically, trypanosome species can be divided into those with large, broad trypomastigotes of the subgenus *Megatrypanum* and those with smaller, slender forms of the subgenus *Schizotrypanum* [49,52]. Most species of bat trypanosomes described to date belong to the subgenus *Schizotrypanum* which, with the exception of *T. cruzi* and *T. rangeli*, appear to be bat-restricted [49]. An important trait of *Schizotrypanum* is that they exhibit intracellular multiplication of amastigote forms [49,52,53].

The significance of bats as dispersers of trypanosome pathogens may be associated with their longevity, habit to agglomerate in large colonies and ability to fly long distances [44]. However, determining the exact role of bats in the transmission cycle of the plethora of *Trypanosoma* spp. they can harbour is challenging. This is because the epidemiology and determinants of cases and outbreaks can be related to the organism pathogenesis associated with a variety of scenarios and peculiarities of local ecosystems, including ecology of susceptible hosts, reservoirs and vectors [44]. For instance, the presence of trypanosome-infected bats in domestic areas could play an important role in bringing parasites such as *T. cruzi* and vectors into closer contact with humans [54]. Another example of the multitude of aspects potentially involved in the transmission cycle of bat trypanosomes is the recent detection of *T. cruzi* in the salivary glands of bats, suggesting that transmission of this parasite to humans could also occur via hematophagous bat bites or salivary contamination of fruits consumed by humans by non-hematophagous bats [55].

Understanding the role bats play in the epidemiology and transmission dynamics of trypanosomes of known medical and veterinary importance, in various sylvatic and domestic ecosystems, is crucial to inform surveillance systems and disease control programs globally. In addition, continuous research on the molecular epidemiology, pathogenicity and zoonotic potential of novel bat trypanosomes is essential to prevent future threats to animal and public health.

In this context, the aim of this review is to synthesise current knowledge on the diversity and epidemiology of bat trypanosomes within a global One Health framework. In particular, the role of bats in the evolution, maintenance and dispersion of known pathogenic *Trypanosoma* spp. in domestic and sylvatic ecosystems will be discussed, with new perspectives on bats’ involvement in their transmission dynamics highlighted. This review also aims at identifying knowledge gaps related to the biology and epidemiology of trypanosomes of bats.

For the purpose of this review, ‘bat trypanosomes’ encompass: (1) bat-restricted trypanosomes (most of which belonging within the *T. cruzi* clade); (2) multi-host trypanosome pathogens (including zoonotic species) known to be infective to bats; and (3) novel *Trypanosoma* spp. that have been described in bats more recently, for which full host-range and pathogenicity are currently unknown.

## 2. Bat Trypanosomes

### 2.1. Diversity, Taxonomy, Distribution and Host Range

The traditional taxonomy of mammalian trypanosomes was proposed by Hoare [5], based on morphology, life-cycle and disease comparisons [5]. Two groups were created based on the mode of transmission and include Salivarian and Stercorarian, which relate to where the infective stage develops within the vector [5]. The Salivarian mode of transmission is based on the development of infective metacyclic trypanosomes within the salivary glands of the vector, with transmission occurring via inoculation. Stercorarian transmission, in contrast, is based on contact with infective forms in the faeces, with the development of metacyclic trypanosomes completed in the hindgut of the vector. Transmission of this type typically occurs when the metacyclic trypanosomes within the faeces penetrate the mucosal membranes around the eye or mouth or via scratches in the skin [5]. For ease of classification, the two-mammalian groups are further divided into different subgenera based on biological features, pathology and epidemiology. Stercorarians are divided into three different subgenera: *Herpetosoma*, *Megatrypanum* and *Schizotrypanum;* while Salivarians are divided into *Duttonella*, *Nannomonas*, *Trypanozoon* and *Pycnomonas* [5]. Only representatives of the subgenera *Herpetosoma*, *Megatrypanum*, *Schizotrypanum* and *Trypanozoon* have been recorded in bats to date, although most species of bat trypanosomes belong to *Schizotrypanum*. Although classification based on the mode of transmission (Stercorarian and Salivarian) proposed by Hoare [5] is highly relevant to trypanosomes and carries historical value, it does not conform to current paraphyletic systems of the genus *Trypanosoma*, or include mammalian trypanosomes that are transmitted via alternative routes [56]. A new approach that keeps with the original morphological descriptions and includes modern phylogenetic approaches has been erected by Kostygov et al. [56]. This proposed classification system still maintains the original mammalian subgenera classification as described by Hoare [5], as they maintain phylogenetic relevance, but also includes newly named subgenera which are explained in detail in the review article by Kostygov et al. [56]. In regard to bat trypanosomes, newly proposed taxonomic calssifications include: (1) the Aneza subgenus comprising *T. rangeli*, *T. vespertilionis* and *T. teixeirae;* (2) the *Trypanosoma wauwau* clade which includes *T. wauwau* and *Trypanosoma madeirae*; and (3) the Australotrypanum subgenus which includes the Australian bat trypanosome *T. vegrandis* [56].

*SchizotrypanumTrypanosoma* spp. are characterised as small bloodstream forms with a mean size range between 14 and 24 μm, including the flagellum. These trypanosomes are distinctively curved with large kinetoplasts that have been described in detail by Hoare [5]. This subgenus comprises both bat-restricted and generalist species that also parasitise a wide range of mammal hosts in South America, Africa, Europe, Asia and Australia [26,37,46,47,54,57,58,59]. Comprehensive phylogenetic studies revealed that the *Schizotrypanum* subgenus forms a monophyletic group, with growing support that representatives of this clade evolved from ancestral bat trypanosomes [26,37,40,49,60,61].

*Trypanosoma cruzi* (the aetiological agent of Chagas disease) is the most iconic and from a One Health standpoint, most relevant species within the *Schizotrypanum* subgenus [5,48]. Biological and phylogenetic relationships based on nuclear ribosomal DNA markers and protein coding genes revealed that *T. cruzi* and closely related bat trypanosomes cluster together to form the *T. cruzi* clade [36,53,54,62]. *Trypanosoma cruzi, Trypanosoma rangeli* and *Trypanosoma dionisii* represent the only generalist parasites within the clade, in contrast to the other related trypanosome sp. which is generally restricted to bats [63].

**Figure 1:**
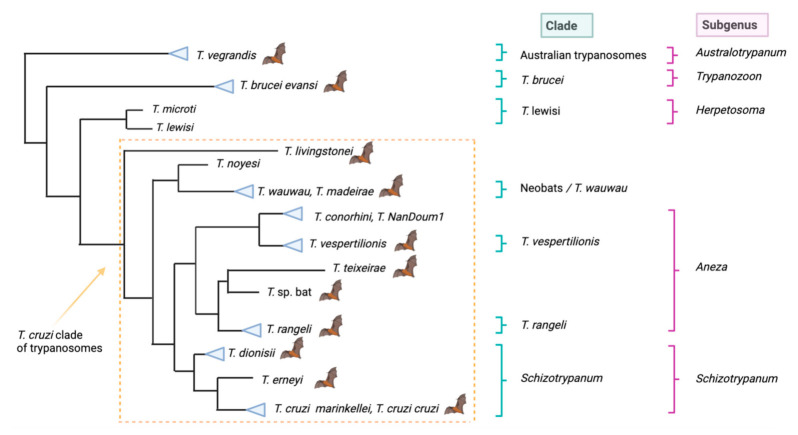
Schematic representation of the phylogenetic relationships shared by bat trypanosomes and corresponding clade and subgenera nomenclature. Bat images indicate *Trypanosoma* spp. of bats. Created with BioRender.com (accessed on 30 August 2021); image not drawn to scale.

Trypanosomes within the *T. cruzi* clade are distributed into three main phylogenetic lineages (Figure 1). The first lineage is the *Schizotrypanum* clade and trypanosomes classified within this clade have morphological similarities to *Trypanosoma cruzi cruzi* (also called *T. cruzi* sensu stricto) [5,49]. This clade consists of *T. c. cruzi* (which is comprised of six Discrete Typing Units (DTUs), TcI–TcVI, and the newly identified genotype Tcbat) [64], *T. cruzi marinkellei,* which is closely related to *T. c cruzi* but restricted to South American bats [40,65], *T. erneyi* hosted in African bats, an undescribed taxon *T*. sp 2 from African bats (Miniopteridae), and *T. dionisii* which has a wide distribution with recent reports in Asian and Australian bats [26,31,46,49,53,57,59,60]. In Australia, *T. dioinsii* was identified in a variety of Australian native microbat species including Gould’s wattled bat (*Chalinolobus gouldii*), the chocolate wattled bat (*C. morio*), the lesser long-eared bat (*Nyctophilus geoffroyi*), the western long-eared bat (*Nyctophilus major*) and the inland broad nosed bat (*Scotorepens balstoni*), demonstrating low host specificity [46]. In China, it has been identified in two bat species, the common serotinus bat (*Eptesicus serotinus*) and Beijing mouse-eared bat (*Myotis pequinius*), while in Japan *T. dionisii* was isolated from the blood of the eastern bent-winged bat (*Miniopterus fuliginosus*) [57,59].

The second lineage, referred to as the *T. rangeli*/*T. vespertilionis* clade or the recently proposed Aneza subgenus, consists of *T. rangeli* which is widely distributed in Central and South America and clusters together with an undescribed trypanosome species (*T*. sp. bat) from African pteropid bats, and with an Australian bat trypanosome, *Trypanosoma teixeirae,* identified in the little red flying fox (*Pteropus scapulatus*) from south-east Queensland, which has a close genetic relationship to *T. rangeli* [56,60,62,63,66,67]. The second lineage also includes *Trypanosoma vespertilionis* from European and African bats and *Trypanosoma conorhini*, a non-pathogenic trypanosome that infects rodents worldwide [59,67]. In addition, undescribed taxon species, *T.* sp NanDoum1 from an African civet (*Nandina binotata*), *T.* sp HochNd1 from an African monkey (*Cercopithecus nictitans*), *T.* sp HochG3 from African bats (*Scotophilus* sp.), *T.* sp Tve-like G1 and *T*. sp Tve-like G2 from African bats and a bat cimicid (*Cacodnus* sp.), respectively, are also included [26,49,56,60].

The third lineage referred to as the Neobat clade or the recently proposed *T. wauwau* clade comprises trypanosome species hosted by Neotropical bats from Central and South America and includes *T. wauwau*, *T. madeirae* and unnamed *T*. sp. Neo bats [56,61]. Interestingly, these species cluster together with *Trypanosoma noyesi,* a species only found in Australian marsupials to date [68]. Basal to the Neotropical group lies *Trypanosoma livingstonei,* which has been reported in Lander’s horseshoe bat (*Rhinolophus landeri*) and Sundevall’s roundleaf bats (*Hipposideros caffer*) from southeast Africa as well as a new subspecies, *T*.cf. *livingstonei,* from Egyptian slit-faced bats (*Nycteris theibaica*) from Africa. Furthermore, an undescribed taxa *T*. sp. 1 from both European and African bats (common bent-wing bat (*Miniopterus schreibersii*) and the Natal long-fingered bat (*M. natalensis*), respectively), also fall within this basal clade [51,60].

*Trypanosoma desterrensis* from the Argentine brown bat (*Eptesicus furinalis*) groups within the *Schizotrypanum* subgenus [34]; however, the use of only spliced leader (SL) RNA locus information to characterise this species prevents its reliable placement within a specific *T. cruzi* subclade, given this locus is not considered an informative phylogenetic marker for long-range evolutionary studies for *Trypanosoma* spp. [33]. This locus, however, is useful for detecting differences between closely related species and demonstrated that *T. desterrensis* has a close genetic relationship to *T. vespertilionis* [34]. A comprehensive list of molecularly characterised bat trypanosomes to date, as well as their host-range, geographic distribution, transmission mode and clinical significance (where known) can be found in Table 1.

Bat trypanosomes have also been reported within the *Megatrypanum*, *Herpetosoma* and *Trypanozoon* subgenera, but to a lesser extent. Trypanosomes within the subgenus *Megatrypanum* are typically characterised as large mammalian trypanosomes that have a kinetoplast situated near the nucleus and far from the posterior extremity of the body. The *Herpetosoma* trypanosomes are medium sized parasites which have a posterior kinetoplast, a pronounced free flagellum and can multiply as either epimastigotes or amastigote forms [5,71]. With the advancement of molecular biology, however, it is not surprising that the original classification of some bat trypanosomes placed into the *Megatrypanum* and *Herpetosoma* subgenera have been challenged. For example, *T. rangeli* was originally placed within the subgenus *Herpetosoma*; however, phylogenetic evidence later revealed a closer relationship to *T. cruzi* than to *Trypanosoma lewisi* (type species for *Herpetosoma*) [58,63]. Furthermore, a comprehensive taxon-rich study showed that *T. rangeli* is more closely related to Old World trypanosomes from rats, bats, monkeys and civets than to *Schizotrypanum* spp. [26]. A new phylogenetic placement was also evident for the rodent trypanosome *T. conorhini* which was originally characterised within the *Megatrypanum* subgenus, but was subsequently reclassified within *Schizotrypanum* [58]. A taxonomy study on the 18S rRNA by Stevens et al. [58], demonstrated trypanosomes classified within the *Herpetosoma* subgenus surprisingly formed a clade together with trypanosomes classified in the *Megatrypanum* subgenus, thus providing evidence that the two subgenera are polyphyletic. Upon these findings the authors proposed that both *Megatrypanum* and *Herpetosoma* subgenera should no longer be used, given the lack of evolutionary or taxonomy relevance to the two groups, demonstrating that phylogenetic studies are critical for species delimitation. In addition, to reduce taxonomic bias it is recommended that a multi gene approach together with whole genome sequencing is used to delineate subgeneric ranks between species of trypanosomes [58,63,72]. Votypka et al. [73] suggested that both traditional and molecular phylogenetics should converge for taxonomic (re)descriptions of Trypanosomatidae and that existing names should be kept whenever possible, especially for medically or veterinary important species. As a result, the present review has adopted the current terminology, which may require further review once a new classification system for Trypanosomatidae has been defined.

Within the *Trypanozoon* subgenus, Central and South American strains of *T. evansi* parasitise *Desmodus rotundus* (the common vampire bat) which acts as a host, reservoir, and biological vector of this parasite. *Trypanosoma vegrandis* hosted by Australian micro bats, has not been assigned to any mammalian subgenus given its small size, unknown biological characteristics, and transmission route. However, Thompson et al. [74] proposed that it should be placed within the Stercorarian group based on its close genetic relationship to the marsupial trypanosomes *Trypanosoma gilletti* and *Trypanosoma copemani* [75,76].

Although the application of molecular techniques has greatly improved the understanding of the diversity and phylogenetic relationships of the genus *Trypanosoma*, a range of bat trypanosomes have been characterised based solely on morphological and parasitological features. Unfortunately, this lack of genetic information has hindered further investigations into the phylogeneny, evolution, diversity and epidemiology of these parasites. Some of these belong to the *Schizotrypanum* subgenus and include: *Trypanosoma pteropi* recorded in *Pteropus* sp. [77], *Trypanosoma hipposideros* hosted by the dusky leaf-nosed bat (*Hipposideros ater)*, and *T. vespertilionis*-like trypanosomes hosted by the black flying fox (*Pteropus gouldii*), [78]. Likewise, *Trypanosoma hedricki* [79] identified in the blood of big brown bats (*Eptesicus fuscus* (Palisot de Beauvois)) and *Trypanosoma myoti* from the blood of the little brown bat (*Myotis lucifugus*) have been classified within the *Schizotrypanum* subgenus based on blood and tissue stages, host specificity and biochemical properties [80].

Approximately 20 species of bat trypanosomes described mainly on morphometric parameters, exist outside the *Schizotrypanum* subgenus, with the majority classified within *Megatrypanum* and *Herpetosoma* subgenera. Limited knowledge is available for these bat trypanosomes, with only a few species successfully cultivated from their bat hosts, including *Trypanosoma incertum* isolated from the common pipistrelle bat (*Pipistrellus pipistrellus*) [32]. Descriptions of the remaining species are based on morphological analysis of the parasite from blood films with the validity of some species uncertain, given the limited number of blood forms and lack of information on life-cycles with the exception of *T. evansi* and *T. vegrandis* which have been molecularly characterised. These bat trypanosomes present globally and are hosted by a variety of bat species as listed in Table 2.

### 2.2. Vectors

Insects are usually involved in the natural transmission of trypanosomes, with success dependent on how effectively the infective metacyclic trypanosomes enter and sustain infection within the vertebrate host. Salivarian and Stercorarian transmission are the two main modes of *Trypanosoma* transmission (as previously mentioned) with the majority of known vectors responsible for the spread of the parasite belonging to the class Insecta [5,36]. Of these, haematophagous insects of the order Hemiptera (e.g., Triatomine bugs and bed bugs), Diptera (e.g., flies), Siphonaptera (e.g., flea) and blood-sucking leeches are considered the main vectors of *Trypanosoma* [5,25,27,38]. However, ticks have also been implemented in the transmission of this parasite [94,95,96,97,98].

Bats are often parasitized by multiple species of ectoparasites, given their social habits and gregarious lifestyle [99]. Most trypanosome-infected bats are insectivorous and often infection occurs through the ingestion of infected arthropods [53]. Bat trypanosomes of the *Schizotrypanum* subgenus are often reliant on triatomine bugs from the Reduviidae family (also known as ‘kissing bugs’) for transmission, with numerous species implicated as vectors. The potential vectors for *T. cruzi* cover more than 130 species of triatomine insects, five of which are epidemiologically more significant: *Triatoma infestans*, *Triatoma brasiliensis*, *Triatoma dimidiata*, *Rhodnius prolixus*, and *Panstrongylus megistus* [100]. *Rhodnius prolixus* and *T. infestans* are considered the principal triatomine vector of *T. c. cruzi* in Central America and South America, respectively [41], while *T. brasiliensis* and *T. dimidiate* are highly domesticated species within northeast Brazil and Central America, respectively [21]. Endemic to Brazil, *Triatoma vitticeps* exhibits high rates of *T. c. cruzi* and to a lesser extent harbours *T. c. marinkellei* and *T. dionisii* [16]. *Trypanosoma c. marinkellei* is also known to be transmitted by *Cavernicola pilosa* and *Rhodnius robustus* as confirmed by xenodiagnosis [26,31,101]. *Trypanosoma rangeli,* on the other hand, is mainly found in the salivary glands of *R. prolixus* with reports of this species also detected in the salivary glands of *T. dimiculata* in Colombia [31,63]. However, complete development of *T. rangeli* only occurs in triatomines from the genus *Rhodnius* [26,29] and unlike *T. cruzi*, *T. rangeli* is considered harmless to its mammalian host [63]. The life-cycle of *T. rangeli* in the triatomine has features representing both the Stercorarian and the Salivation life-cycles, with trypanosomes present at both the anterior and posterior ends. Before the advent of molecular biology, the taxonomic status of this trypanosome was a subject of controversy, given the combination of distinct transmission routes [102]. Phylogenetic studies helped to resolve this and, as mentioned in Section 2.1, analysis at the 18S ribosomal RNA locus revealed a close taxonomic relationship to *T. cruzi* and *T. dionisii,* placing *T. rangeli* into the *Schizotrypanum* subgenus [58,63,103]. Cimicid (Cimicidae) bugs—commonly referred to as ‘bed bugs’ or ‘bat bugs’—have also been shown to transmit bat trypanosomes [18,32,40]. Cimicid bugs are frequently found infected with trypanosomes with reports of *T. dionisii*, *T. vespertilionis* and *Trypanosoma incertum* identified in *C. pipistrelle*, *T. hedricki* identified in *C. brevis,* and *T. cruzi* found in *C. lectularius* [32,79,80,104]. Furthermore, *T. vespertilionis-*like G2 which is similar to the European *T. vespertilionis* P14 genotype, has been found in the gut of a bat cimicid (*Cacodnus* sp.) from Africa [26]. In addition, a *Trypanosoma* sp. with *Schizotrypanum* characteristics was described in a bat bug (*Stricticimex brecispinosus*) from Africa [60,105]. Rodhain [106] showed the development of metacyclic trypanosomes in the gut of *C. lectularius* after they fed on cultures of *T. vespertilionis* and concluded that transmission was contaminative. Gardner and Molyneux [32] also successfully demonstrated infection of *C. lectularius* with *T. incertum* but were unable to prove transmission due to negative xenodiagnosis, which is a method that exposes potential infected biological material to uninfected vectors which are subsequently examined for the presence and development of pathogens. A study by Liao [90] reported the successful experimental transmission of *T. scotophilus* into greater Asiatic yellow captive bats (*Scotophilus heathi*) via the ingestion of infected *C. lectularius* and through skin abrasions contaminated with the hindgut contents from the bugs containing metacyclic trypanosomes [32].

In addition to bugs, sand flies that bite bats have been implicated as trypanosome vectors, with *Trypanosoma pessoai* believed to be transmitted by *Dipteran* sp. [69]. It has been speculated that the *T. pessoai* flagellates detected in *Phlebotomus vespertilionis* are transmitted to the vertebrate host via contamination in superficial skin lesions or bite wounds, given the absence of metacyclic trypanosomes detected in the anterior station of the insect [107].

The vectors for bat trypanosomes in Africa and Australia are currently unknown. Polyctenidae bat bugs are commonly associated with African bats and have been suggested as potential vectors for *T. erneyi* [49]. In Australia, bat flies (Nycteribiids) may act as vectors for Australian *T. dionisii,* as these insects are obligate hematophagous ectoparasites that play important roles in the transmission and maintenance of bat pathogens and are commonly found cohabitating with Australian bat species [46,99].

**Figure 2:**
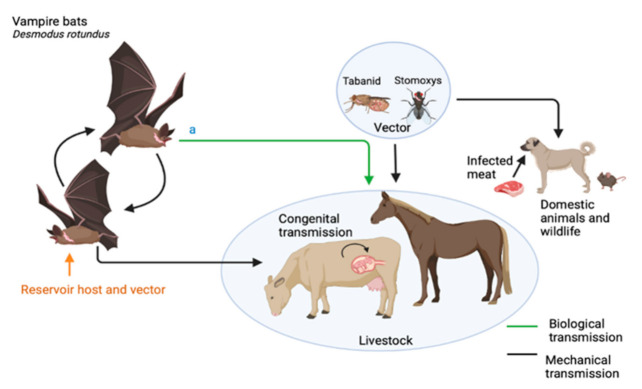
Transmissional route scenarios adapted by *Trypanosoma evansi.* **^a^** In South and Central America, biological systems for the transmission of *Trypanosoma evansi* have been established in vampire bats (*Desmodus rotundus*). Created with BioRender.com (accessed on 5 August 2021); image not drawn to scale.

*Trypanosoma evansi* is mainly transmitted by hematophagous biting flies of the genera *Tabanus* and *Stomoxys* [6]. In contrast to biological transmission of several other *Trypanosoma* spp. of the *T. brucei* complex by tsetse flies (genus *Glossina*), transmission of *T. evansi* through *Tabanus* and *Stomoxys* is mechanical [5] with various routes of transmission depicted in Figure 2. *Trypanosoma evansi* can also be transmitted to mammalian hosts by bats (as also represented in Figure 2). This biological mode of transmission of this parasite is further discussed in Section 2.4.

### 2.3. Life-Cycle and Pathogenesis

Trypanosomes have a complex life-cycle which generally involves an invertebrate and a vertebrate host, although transmission from one mammal host to another may occur (see Section 2.4). Parasite development in the invertebrate host often involves several cycles of multiplication and differentiation in the digestive tract, and migration to various developmental sites resulting in Salivarian (e.g., *T. rangeli)* or Stercorarian (e.g., *T. cruzi*) transmission [5]. As discussed, *T. evansi*, although genetically most similar to *T. brucei*, has adapted to mechanical transmission in biting flies, which depends on the survival of the parasites in the oral cavity of these vectors [5].

The life-cycle of *Trypanosoma* in the vertebrate host varies from one subgenus to another and may involve different stages of the parasite. This variability partly lies in the capability of some species to invade and multiply within host cells (amastigote forms), and then differentiate to trypomastigotes (non-dividing form), which are released in the bloodstream upon rupture of the cells. Trypomastigotes then infect other cells and are eventually disseminated throughout the body via the lymphatic system and bloodstream, leading to the establishment of the infection in several host tissues [108]. This is the case for *T. cruzi* and other phylogenetically closely related bat trypanosomes such as *T. dionisii* and *T. erneyi* [49,51,109].

The biological mechanism(s) of invasion by *Trypanosoma spp.* and the associated regulatory pathways involve several mammalian host cell receptors [110]. In *T. cruzi* infections, the most commonly infected cells include cardiac myocytes, endothelial cells, peripheral skeletal and smooth muscle cells, and cells of the nervous system [111]. Tissue inflammation caused by the presence of intra-cellular *Trypanosoma* life forms may cause severe chronic symptoms in the host which is maintained by intracellular propagation of the parasites in the smooth muscles and other host tissues and may lead to myocardial failure and death [112,113]. In addition, the acute phase of *T. cruzi* infection may be associated with anaemia, thrombocytopenia, leukopenia, and bone marrow hypoplasia in mammalian hosts [114]. The mechanisms of anaemia are thought to be induced by the innate immune response, which leads to haemolysis of infected cells, or by haemolysins released by the parasites [115,116]

The high intra-specific genetic variability of *T. cruzi* has implications for the pathogenesis of Chagas disease, as specific outcomes may be determined by parasite genetics and interactions in mixed infections [117]. Additionally, the potential effect of host genetics on the expression of parasite virulence and the outcome of infection in both laboratory induced and naturally occurring *T. cruzi* infection studies require further investigation [118].

In contrast to *T. cruzi* and other closely related *Trypanosoma* spp., the replication of trypanosomes belonging to the *T. brucei* complex (which includes *T. evansi*) in the vertebrate host occurs extracellularly in peripheral blood, where they transform into trypomastigotes. Importantly, this life-cycle stage has also been found in extravascular sites of lymph nodes, kidney, spleen, bone marrow and brain [5].

Knowledge on the life-cycle and potential pathogenicity of trypanosomes in bats, including how bat immune responses interact with parasites known to cause disease in other hosts, is very limited [119]. Evidence to date strongly suggests that there is no association between clinical disease and trypanosome infection in bats, with only one case of trypanosomiasis in bats documented to date [120]. The disease was caused by *T. teixeirae*, a novel species belonging to the *T. cruzi* clade, in an Australian little red flying fox (*Pteropus scapulatus*). The bat exhibited high parasitaemia and acute clinical signs including anaemia, patchy haemorrhage, icterus and histiocytic inflammation [47,120]. The authors, however, highlighted that trypanosomes may occur in very large numbers in the blood of animals suffering from a primary immunosuppressive disease.

The fact that most trypanosomes of bats appear to be benign to these flying hosts is important from an epidemiological perspective, as not succumbing to infection maximizes reservoir competency. Another relevant factor associated with the role of bats in maintaining and dispersing *Trypanosoma* spp. across various ecosystems is that these animals exhibit long lifespans, and therefore infection may last for years, with trypanosomes residing in skeletal, cardiac and abdominal muscles [121].

### 2.4. Alternative Transmission Routes

Although trypanosome transmission is generally associated with vectors, alternative modes do occur as often seen with *T. cruzi*. Normally this parasite is transmitted through the faeces of triatomine bugs, contaminating their own bite wound on the vertebrate host’s skin. Alternative routes include congenital transmission, contamination through blood transfusion and organ donations, consumption of contaminated material, or predation on infected hosts or vectors [44]. Another interesting mode of transmission involves Didelphid marsupials which may infect other mammals through contaminated secretions from their scent glands, which are released in situations of stress [44]. These alternative routes of transmission are impacting the epidemiology of trypanosomes, especially in today’s modern world. Once confined to South America *T. cruzi* is now being reported globally. This is due to increased human travelling and migration, and transmission via non-vectorial routes, including the transmission of contaminated blood products and organs as well as congenitally [122]. In addition, the re-emergence of *T. cruzi* in previously non-endemic regions can be attributed to the consumption of contaminated food sources via oral transmission [18]. This mode of transmission is not a recent one, as numerous studies have confirmed the ingestion of infective stages of *T. cruzi* may lead to the development of Chagas disease in the host [108,123,124]. The first outbreak of orally transmitted Chagas disease affecting humans was reported in Brazil in 1968 [125] due to a shared contaminated meal [126]. Since then, several outbreaks have occurred in the State of Santa Catarina in Brazil (2005) and in the Brazilian Amazon basin (between 1982 and 2001) due to the consumption of infected Amazonian palm berry or açai (*Euterpe oleracea*) juice. In a recent outbreak of acute Chagas disease induced by oral transmission, 128 students from a municipal school in Venezuela tested positive with one incident of death reported. The outbreak was linked to the consumption of freshly prepared fruit juice that was prepared in a triatomine infested area. Consequently, new guidelines into the prevention, management, and diagnosis of acute Chagas disease were implemented by the national Ministry of Health [127]

Vampire bats can transmit trypanosomes directly by mechanical mechanisms via the transfer of trypanosomes from an infected host or a contaminated substrate to a susceptible host. For example, *Trypanosoma hippicum*, *T. equinum*, *T. theileriae*, *T. rangeli*, *T. dionisii*, *T. pessoai*, *T.madeirae*, *T. c. marinkellei*, and *T. c. cruzi* have been recorded in common vampire bats (*Desmodus rotundus*) which facilitates the spread of these parasites directly by contaminated mouthparts [5,19,20,42,128]. The social behaviour and cooperative relationships of bats with grooming (ingestion of trypanosome infected ectoparasites) and regurgitated blood sharing, may also help facilitate the mechanical transmission of these bat trypanosomes [26,129]. In addition, vampire bats have a preference for house dwellings, and there are many reports of humans being bitten, including 154 cases of vampire bat bites, over several months in a single Venezuelan village [130]. Ramirez et al. [70] reported a high prevalence of *T. cruzi* in *D. rotundas* in Eastern Colombia and thus infected bats are considered a significant risk for trypanosome transmission, particularly in areas where bat–human interaction is frequent. Furthermore, a surveillance study to ascertain the occurrence of *T. cruzi* in bats within the Peruvian Amazon Basin, reported the first incidence of *T. cruzi* in the salivary glands of a hematophagous bat *Diaemus youngi* (white winged vampire bat) [55]. These findings suggest *D. youngi* may be a mechanical vector of Chagas disease, via salivary contamination [55]. A follow up study by Bergner et al. [131] examined repurposed viral metagenomic data for detection of *T. cruzi* in bat saliva. Data analysis found TcI in the saliva of two vampire bat species (*D. rotundus* and *Diphylla ecaudata*) captured in Peru and supported the findings by Villena et al. [55]. Further investigations including transmission studies are however needed to evaluate the putative role of bats as vectors of *T. cruzi*.

The vampire bat *D. rotundus* can also act as biological vector of *T. evansi* in South America. In this unusual transmission mode, after ingestion of contaminated blood from a prey (most often horses, cattle) or other bats, infective forms of the parasite can be found in the bats’ saliva and passed on to the next host via bat bite or licking [5,20]. The epidemiological relevance of bats as reservoirs and vectors of *T. evansi* and associated One Health implications are further discussed in Section 3.3.

### 2.5. Atypical Cases of Animal Trypanosomiasis in Humans

Atypical cases of animal trypanosomiasis have been reported in humans including infants being infected with *T. lewisi* from Southeast Asia and Africa and *T. evansi* causing human trypanosomiasis in an Indian farmer who suffered fluctuating parasitaemia with repeated febrile episodes for a duration of five months [132,133,134]. Transmission of *T. evansi* was speculated to be a result of contamination from the blood of an infected animal onto an open wound on the man’s skin [135] thus highlighting the need to better understand alternative transmission routes of *Trypanosoma*.

*Trypanosoma dionisii,* which was believed to be restricted to bats, has been detected in human cardiac tissue together with four *T. cruzi* DTUs (TcI-TcIV) in a 2-year-old patient who had acquired acute Chagas disease via the oral route. The patient subsequently died and the handling of a recently dead (and infected) reduviid bug (*T. vitticeps)* was believed to be the cause of the *T. cruzi* infection [18,135]. *Trypanosoma dionisii* is considered non-pathogenic; however, experimental studies by Oliveira et al. [109] reported the replication of *T. dionisii* in mammalian cells in vitro. Additionally, Gardner and Molyneux [32] found *T. dionisii* amastigote stages in thoracic skeletal muscle from the common pipistrelle microbat (*Pipistrellus pipistrellus).* Collectively, these findings suggest *T. dionisii* may potentially adversely affect the health of its mammalian hosts. Further investigations of the clinical impact of *T. dionisii* are warranted, especially given its global distribution and recent evidence of lack of bat host specificity.

### 2.6. Parasite Detection Methods

For many years, before the advent of molecular technologies, detection of *Trypanosoma* spp. was based solely on direct observation of the parasite in blood smears by light microscopy [5]. However, microscopy lacks sensitivity, particularly in chronic stages of infection and species descriptions based exclusively on morphological characterisation may be confounded by overlapping morphometry among species and polymorphic life-cycle stages in vertebrate hosts [74,136,137].

Since the late 1980s, PCR of blood or cultured parasites at multiple loci, Sanger sequencing of amplicons and phylogenetic analyses, coupled with traditional and advanced microscopy techniques, have uncovered a broad genetic diversity of *Trypanosoma* spp. in a range of mammals, including bats, globally (e.g., [47,49,51,54,64,138]).

Overall, sequence analysis of partial fragments of the 18S rRNA locus is the most widely used method to screen wildlife samples, including bats, for *Trypanosoma.* However, amplification at additional loci such as the nuclear glyceraldehyde 3-phosphate dehydrogenase gene (gGAPDH) and mitochondrial cytochrome (Cytb) gene, provide a more robust genetic characterisation [38,54,68].

Multi-locus typing applied mostly to the study of cultured parasites has enabled numerous investigations of intra-specific *T. cruzi* diversity, in both single and mixed infections [64,139]. Indeed, a large repertoire of DNA targets (e.g., kDNA, intergenic region of spliced leader genes (SL-IR), rDNA 24sα, ITS1 rDNA) and a variety of typing tools (e.g., real-time PCR, multiplex PCR, Restriction Fragment Length Polymorphism (PCR-RFLP), Random Amplification of Polymorphic DNA (RAPD), DNA hybridization and karyotyping) have been explored in *T. cruzi* molecular investigations [54,112,140,141,142,143,144]. Multi-locus genotyping is considered the gold standard method in Chagas disease population studies as no single genetic target allows complete DTU resolution [64].

Although the most widely used sources of DNA for molecular detection of *Trypanosoma* are whole blood, serum and cultured parasites, in a recent study, a PCR approach targeting the 18S rDNA in blood clots proved more sensitive when compared to buffy coat and whole blood, when evaluating the diversity of trypanosomes infecting bats, canids and marsupials [145]. The authors highlighted that this method is particularly advantageous when investigating trypanosomes of bats which have a small body mass and, consequently, low blood volume [145].

An important limitation of direct parasite detection methods is that reproducibility and overall performance of PCR-based methods can be significantly affected by the fluctuations in parasitaemia during the chronic phase of infection [146,147]. Another issue is the high prevalence of *Trypanosoma* polyparasitism (i.e., the simultaneous presence of two or more *Trypanosom*a sp. within the same host), which appears to be a widespread phenomenon in a wide range of vertebrates hosts (fish, amphibians and mammals), and invertebrate vectors) [148,149,150,151,152,153,154,155]. In light of this, efficient and reliable methods to audit bat trypanosome communities within each individual host are crucial not only for estimating the diversity and prevalence accurately, but also to unravel the interactions (either synergistic or competitive) amongst these organisms.

A number of more traditional molecular strategies can be used to identify mixed infections in bats and other hosts (e.g., cloning and species-specific PCR) [54]. Next-generation sequencing (NGS) technology is however superior at auditing parasite diversity and prevalence of co-infection, without a priori hypothesis. A novel 18S rDNA NGS metabarcoding methodology was recently developed and used to characterise *Trypanosoma* co-infections in Australian marsupials and their ticks [149,156] and Brazilian bats [149,156,157]. Not surprisingly, these studies revealed a high prevalence and diversity of trypanosome co-infections and uncovered novel genotypes which were present in a lower abundance. This high-throughput technology may prove extremely valuable in future studies of bat trypanosome epidemiology, ecology and evolution.

## 3. Epidemiology and Evolution of Bat Trypanosomes in a One Health Framework

### 3.1. Role of Bats in the Evolution of Trypanosoma cruzi: The Bat Seeding-Hypothesis

Given its zoonotic nature and pathogenicity to humans, *T. cruzi* has been studied extensively and has been the subject of debate particularly its evolutionary origins. Phylogenetic studies based on 18S rRNA and gGAPDH loci have been used to address evolutionary and taxonomic questions related to the genus *Trypanosoma*, with focus on the *Schizotrypanum* subgenus which have been reported in all continents in which bats are found [37,40,54,60,62]. In 2012, Hamilton et al. [40] proposed the bat seeding hypothesis after extensive phylogenetic studies on *Trypanosoma* based on the 18S rRNA, gGAPDH and Cyt b regions, resulting in the formation of a monophyletic assemblage containing species within the *T. cruzi* clade [36,38,40,49,53]. Hamilton et al. [40] hypothesised that bats were the ancestral hosts of the *T. cruzi* clade and given their ability to disperse over large areas, cross oceans and continents, bats dispersed their ancestral trypanosomes into terrestrial mammals and marsupial host over time, resulting in new linages within the clade.

Follow up studies based on multilocus phylogenetic analyses provided additional support for the bat-seeding hypothesis, with the placement of trypanosomes from bats (*T. erneyi* and *Trypanosoma sp*. HochG3) and a bat bug (*Cacodnus* sp) from Africa, within the *T. cruzi* clade [26,49]. Additional studies into bat trypanosomes from Australia and Asia also corroborated the hypothesis with the placement of *T. teixeirae* and *T. dionisii* isolated from Australian bats within the *T. cruzi* clade, together with *T. dionisii* identified in bats from China and Japan [46,47,57,59]. Interestingly, Espinosa-Alvarez et al. [26] reported close genetic similarities of African bat (*Scotophilus* sp.) trypanosome genotypes *T.* sp HochG3 to a *T.* sp HochNdi1 from an African monkey (*Cercopithecus nictitans*), and the adaptions of ancestral bat trypanosomes, to diverge and establish into new terrestrial hosts with old world cimicids, suggested as the likely vector.

The different geographical origins of the *Trypanosoma* species that make up the *T. cruzi* clade, is likely due to the migration of bats between the New and Old Worlds as suggested by Hamilton et al. [40] and may account for the present distribution of closely related and recently diverged bat trypanosomes [49,60]. The close genetic similarities between *T. erneyi* (Africa) to both *T. c. marinkellei* and *T. cruzi* (American), *T*. sp HochG3 (Africa) to *T. vespertilionis* (Europe), South American *T. wauwau* and African *T. livingstonei* to Australian marsupial species including *T. noyesi* as well as the worldwide distribution of *T. dionisii* (Europe, Africa, Asia, Central America, Australia) may be explained by the intercontinental range of bats [26,35,49,60].

A recent geographical survey to underpin the origins and ancestors of the *T. cruzi* clade by Clement et al. [60] reported a high prevalence of *T. cruzi* clade species (*T. dionisii*, *T. vespertilionis*) in African bats, as well as novel trypanosome species *T.* sp 1 genetically similar to *T. livingstonei* from Africa in European bats (*M. schreibersii*). Based on these findings, together with ancestral area reconstruction and biogeographical analysis, Clement et al. [60] provided strong evidence that most trypanosomes from the *T. cruzi* clade have an African origin, and that trypanosomes of the *T. cruzi* clade have radiated from Africa through several dispersion events, to other continents throughout the world. Further geographical surveys to include bat trypanosomes from other regions of the world, especially Australia and Asia have been suggested to enable resolution of taxonomic issues within the *T. cruzi* clade [60].

### 3.2. The Complex Diversity and Epidemiology of Trypanosoma cruzi of Bats

**Figure 3:**
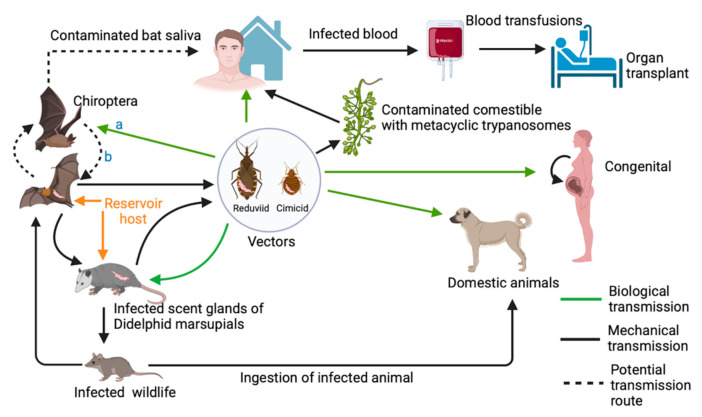
Transmissional route scenarios adapted by *Trypanosoma cruzi*. **^a^** Transmission of trypanosomes by the vector during a blood meal or ingestion of the whole vector by the Chiroptera. **^b^** Potential transmission of trypanosomes among bats via regurgitated blood sharing or ingestion of trypanosome infected ectoparasites during grooming. Created with BioRender.com (accessed on 26 August 2021); image not drawn to scale.

As previously mentioned in Section 2.1, the species complex *T. cruzi* is divided into two subspecies: *T. c. cruzi* and *T. c. marinkellei* [30]. Whilst *Trypanosoma c. marinkellei* is a bat-restricted parasite, *T. c. cruzi* (hereafter referred to as *T. cruzi* for simplicity) is a generalist, phenotypically and genotypically diverse zoonotic pathogen, present in enzootic cycles in the southern US through to southern South America [64]. The transmission cycles of *T. cruzi* are complex networks that may involve humans, multiple haematophagous arthropod species and over 70 genera of mammalian reservoir hosts, including a wide range of neotropical bat species [54,64,158]. In addition to playing key roles as parasite reservoirs, these non-human hosts may also serve as complex selective systems leading to the emergence of novel parasite traits [159]. A general schematic representation of known *T. cruzi* transmission cycles and the corresponding roles played by bats is represented in Figure 3. It is important to highlight, however, that particularities of each endemic ecotope must be taken into consideration when interpreting the image.

In humans, Chagas disease is considered to be one of the most important parasitic diseases in Latin America with a serious impact on public health and national economies. This zoonosis primarily affects poor rural populations and its distribution is constantly influenced by human activities, resource utilization, and other extrinsic environmental factors [26].

From a global health standpoint, migratory trends of infected populations from rural to urban areas as well as to non-endemic regions, along with changes in the ecology and distribution of vector populations, have led to the gradual urbanization and globalization of Chagas disease, which is now recognized as an emerging threat to public health worldwide [160,161]. To date, Chagas disease has been detected in non-endemic countries from North America (Canada and the U.S.), Europe (mainly Spain), and the Western Pacific Region (Australia, New Zealand, and Japan) [162].

Hence, this protistan parasite is a pathogenic agent for which evolution and population structure are among the best studied, although not necessarily the best understood [64].

#### 3.2.1. The Repertoire of *Trypanosoma cruzi* Genotypes in Bats

Collectively, research efforts involving bats from a range of domestic and sylvatic ecosystems in the Americas and other non-endemic areas for Chagas disease, have shown that bats can harbour five (TcI–TcIV and Tcbat) out of the seven *T. cruzi* DTUs described to date [40,49,51,53,54,61,121,128,163]. Studies have also revealed that the epidemiology of Chagas disease in both human and animal populations may vary across endemic areas, with DTUs linked to preferential ecological niches within domestic and/or sylvatic environments, as well as different transmission patterns and clinical manifestations [44,160].

Bats naturally infected with *T. cruzi* TcI strains have been predominantly observed in sylvatic cycles in the Andes region and in Central and North America [64,164,165]. However, TcI has also been associated with human infections reported from the US [166], Mexico, Central America [167], and northern South America [104,144,168,169,170]. Interestingly, among TcI isolates, marked genetic subdivision has been observed between strains from domestic and sylvatic transmission cycles, largely independent of geographic origin. Clinical presentations of TcI include chagasic cardiomyopathy and in immunocompromised hosts severe cases of meningoencephalitis [64]. Trypanosoma *cruzi* TcIII, similarly, is mostly associated with the sylvatic cycle albeit it is distributed further south in Brazil and adjacent countries. Documented human infections involving TcIII are rare; however, this genotype has been occasionally found in domestic dogs in Paraguay and Brazil, and in peridomestic *Triatoma rubrofasciata* in southern Brazil, which may indicate that this strain may yet become more prevalent in humans [64].

The bat-infective strains TcII and TcIV, dominate in domestic cycles. Along with TcV and TcVI (which have not been recorded in bats), TcII is the main causative agent of Chagas disease and is found predominantly in the southern and central regions of South America, particularly in eastern and southern parts of Brazil [54,64]. From a clinical perspective, TcII has been associated with cardiac manifestations, megaesophagus and megacolon [64]. TcIV exhibits a similar geographic distribution in South America to TcIII, with the exception of the Argentine Chaco region, where it has not been recorded to date. However, unlike TcIII, TcIV occurs fairly frequently in humans and is a secondary cause of Chagas disease in Venezuela [171]. Interestingly, TcIV has been identified in primates and assassin bugs (*Rhodnius brethesi)* in the Amazon basin, indicating that this parasite strain can have an arboreal ecotope [172,173].

Research aiming to unveil the complex zoonotic cycles of *T. cruzi* through investigations of the genetic diversity of *T. cruzi* in bats has also uncovered a distinct *T. cruzi* DTU (named Tcbat) in a range of bat species from Colombia [54,70,172] and Panama [128]. *Trypanosoma cruzi* Tcbat was subsequently detected in a child from Colombia and mummies from Chile [174,175].

Mixed infections involving multiple *Trypanosoma* spp. and/or *T. cruzi* DTUs are very common in the wild, particularly in chiropterans and marsupials [44]. However, the epidemiological and clinical implications of these intra and inter-specific parasite interactions constitute a current knowledge gap and therefore require further investigation.

#### 3.2.2. Bats as Reservoirs of *Trypanosoma cruzi* in the Domestic and Sylvatic Cycles

Chagas disease was initially an endemic disease of animals (enzooty) maintained among wild animals and vectors, which was transmitted accidentally to humans when they entered sylvatic ecosystems. This still occurs in areas from the southern US to southern Argentina and Chile. Over time, human encroachment and constant deforestation has led to closer contact with wild reservoirs of the disease. Most importantly, triatomine vectors have adapted to peridomestic areas, forming endemic areas in South and Central America and in Mexico [176].

The epidemiological significance of bats as reservoirs of *T. cruzi* in specific ecosystems across endemic areas for Chagas disease depends on a variety of factors relating to host-vector-parasite relationships. Bats are known to acquire *T. cruzi* infections during the blood meal of an infected vector (e.g., triatomines) or through the ingestion of these arthropods. Bats can encounter triatomines during foraging and feeding at night when both are active, as well as potentially being fed upon by triatomines during the day when roosting in trees or caves [177].

The degree to which bats maintain *T. cruzi* parasitaemias and thus are infectious to vectors has not been well-elucidated; however, the isolation of *T. cruzi* from the blood of bats in Central and South America supports their status as reservoirs in those areas [49,53,128]. Interestingly, congenital transmission of *T. cruzi* from pregnant female bats (*Molossus molossus*) to their foetus has been reported in western Venezuela [158]. This finding indicates an additional mechanism supporting the maintenance of this zoonotic pathogen in the environment.

Sylvatic cycles of *T. cruzi* transmission consist of numerous, complex and relatively poorly understood niches with different eco-epidemiological properties, each one involving multiple sylvatic and/or synanthropic triatomine species, which in turn feed on a variable range of rodents, primates, carnivores, bats, marsupials (i.e., opossums) and xenarthrans (i.e., armadillos, sloths, anteaters) [64,159]. In this scenario, *T. cruzi* DTUs circulate in relatively independent sylvatic cycles with particular ecological niches and preferentially or opportunistically determined mammals and vectors. However, members of the same DTU are also known to infect mammals of distinct species and orders, indicating that host-switching may be common among sympatric hosts [172,173,178,179].

A comprehensive *T. cruzi* survey of free ranging wild mammalian fauna from five biomes of Brazil revealed that overall, opossums demonstrated higher rates of positive hemocultures, whereas Chiroptera were distinguished for hosting the greatest diversity of species and genotypes of *Trypanosoma* spp. [44]. The authors of the study, however, highlighted that no generalisation should be made and each ecotope should be considered as a unique system when it comes to establishing control and education measures for the local population [44]. Another interesting study assessed a possible association of trypanosome prevalence with habitat fragmentation in bats in sylvatic settings and found a greater prevalence in bats from areas with fragmented forests than in bats from regions of continuous forests [61].

The transmission dynamics of *T. cruzi* have been more extensively investigated in domestic environments compared to sylvatic settings (reviewed in [176]). This is likely due to the Public Health significance and the relative ease of sampling in domestic and peridomestic areas. Research has shown that bats roosting in man-made structures, including buildings, dwelling lofts and thatched roofs, can harbour *T. cruzi* infection. Thus, these bats can play an important role in the distribution and transmission dynamics of trypanosomes by bringing *T. cruzi* into closer contact to humans [54]. This scenario is further supported by the fact that several species of triatomine vectors (i.e., *T. infestans*, *T. dimidiata*, *T. brasiliensis*, *Triatoma pseudomaculata, Triatoma sordida, Triatoma maculata, Rhodnius ecuadoriensis*, *R. prolixus, R. pallenscens*, *Pastrongylus geniculatus* and *P. megistus*) have adapted to live in domiciliary settings and to blood-feed on humans and/or domestic animals (which can also become infected with *T. cruzi*) [176,180]. Moreover, in addition to being regularly found in domestic animal shelters, triatomine vectors may also live in bat nests in domestic and peridomestic areas [54]. Mejia-Jaramillo et al. [181] reported that some of the main risk factors for Chagas disease transmission in domestic settings are: (1) high infection rates of people and domestic animals, (2) the presence of infected triatomines inside the human dwellings and (3) the proximity between houses and wild animals.

Although experimental trials have shown that some triatomine species can have strong preferences for a particular host when given a choice [180,182], subsequent studies have supported a scenario where *T. cruzi* vectors have adapted to multiple blood-feeding sources, which facilitates its transmission within and between sylvatic and domestic cycles [176,183]. In agreement with this, a recent study in Colombia conducted an amplicon-based NGS characterization of Triatominae feeding sources (i.e., *Panstrongylus*, *Rhodnius* and *Triatoma*), and found multiple domestic and sylvatic vertebrate feeding sources (including chiropterans) per triatomine [183]. Another study obtained experimental evidence that *R. prolixus* can feed upon *Carollia*, *Glossophaga*, and *Desmodus* bats, suggesting that frequent transmission of *T. cruzi* between bats and humans via triatomine vectors is feasible [183,184]. Moreover, many bat species have omnivorous feeding habits and can feed on small mammals and triatomines, which means the probability of infection with *T. cruzi* could be slightly higher for these animals [183].

Collectively, these findings provide further evidence of how bats may be playing a significant role in the epidemiology of *T. cruzi* in the New World tropics and shed more light into how *T. cruzi* occasionally moves from sylvatic settings to initiate new domestic and peri-domestic transmission cycles of Chagas disease.

### 3.3. Epidemiological Role of Bats as Reservoirs and Vectors of Trypanosoma evansi

*Trypanosoma evansi* exhibits a wide spectrum of virulence levels in different host species with multiple clinical symptoms, indicating the presence of diverse reservoirs and complex epidemiology. A recent systematic review of the global distribution and host range of *T. evansi* revealed that this pathogen is distributed across 48 countries in Africa and Asia, seven countries in South America, and four in Europe (imported cases only) [6]. The authors highlighted, however, that this is an underestimation as many countries do not report surra cases to the World Organisation for Animal Health (OIE), even though it is a notifiable disease. The research also confirmed that *T. evansi* infects a wide range of domestic and wild animals in Africa, Asia and South America with the highest prevalence observed in dromedary camels (in Africa and the Middle East) followed by water buffaloes, cattle, dogs and horses in East and Southeast Asia. In South America, the acute form of the disease was reported in horses and dogs [6].

While insectivorous, nectarivorous and frugivorous bats generally have little importance in the *T. evansi* enzooty [5], haematophagous bats are implicated in the transmission cycle of *T. evansi* as reservoirs and biological vectors (when they do not die due to clinical signs of surra after infection). Although the vectorial role of vampire bats is epidemiologically relevant in horses and cattle in South America, transmission of *T. evansi* via biting flies is still the most important mode of transmission and maintenance of the surra enzooty in this continent and all other endemic areas [20]. Further studies are required to determine the prevalence of *T. evansi* in a range of bat species and other reservoir hosts to better quantify the relative risk these animals pose as both reservoirs and vectors of this pathogen in a One Health context.

Whilst *T. evansi* is the only member of the *Trypanozoon* subgenus that is recognised as a ‘bat trypanosome’, there is one anecdotal report of successful infection of *T. brucei* in fruit-eating bats (Megachiroptera) and insect-eating bats (Microchiroptera) [50]. Based on this result and since some species of tsetse flies (*Glossina*) are known to feed on bats, the authors raised the possibility of insect-eating bats acting as potential reservoirs of this important zoonotic pathogen [50]. To the best of our knowledge, to date no follow-up studies have been conducted to confirm this hypothesis.

## 4. Biosecurity Concerns

Biosecurity concerns associated with exotic bat trypanosomes have been mainly discussed in Australia, which has a unique native fauna which has been shown to be susceptible to the negative impacts of *T. evansi* [10] and possibly *T. cruzi* [185,186]. This is because *T. cruzi* is genetically closely related to the Australian *T. noyesi* [68,187]. Thus, it has been hypothesized that the vector(s) of *T. noyesi* could potentially transmit *T. cruzi* from humans (infected immigrants and travelers, or possibly Australians who contracted the infection via blood transfusion) to indigenous mammals [68,185]. Experimental *T. cruzi* infections confirmed the potential for disease and mortality of some native mammals, as well as their potential to act as a reservoir for human infection [185,188]. In this scenario, naïve Australian bat populations could also be infected and their health negatively impacted. Additionally, some bats could develop lifelong chronic infections and become key reservoirs in newly established transmission cycles. These hypotheses could also potentially be extrapolated to non-endemic continents such as Africa and Europe, where bats also harbour *Trypanosoma* spp. with a genetically high similarity to *T. cruzi* [49,51,54]. Continued surveillance of trypanosomes in bats and other wildlife in these areas is crucial to monitor the potential biosecurity risks in the future.

## 5. Concluding Remarks and Future Directions

This review brings together scientific evidence that supports a large diversity of *Trypanosoma* spp. harboured by chiropterans. To date, 37 bat trypanosome species have been formally recognised and numerous genotypes identified in bats, revealing a higher inter and intra-specific genetic diversity than previously anticipated. Molecular studies have also significantly aided our understanding of the complexities associated with the biology, ecology, phytogeography and evolution of these parasites, with key findings outlined in this article. Despite these remarkable advances, it is a consensus in the literature that the prevalence and diversity of trypanosomes in bats are still likely underestimated. Moreover, there is a large knowledge gap concerning the vectors, life-cycle and host-range of most bat trypanosomes recorded to date, particularly those not known to be zoonotic and/or pathogenic.

As highlighted in this review, the vast majority of bat trypanosomes are morphologically and genetically most similar to *T. cruzi*. Whilst evolutionary studies suggest that bat trypanosomes of different genetic backgrounds lacked host specificity, and successively adapted to a range of mammalian hosts, the mechanisms that allowed these parasites to cross host species barriers and emerge as human pathogens are unknown. In this framework, the emergence of *T. dionisii* as a potential human pathogen represents an example of how bat-restricted trypanosomes within the *T. cruzi* clade could infect and cause disease in humans. From a One Health viewpoint, it is also crucial to investigate and monitor the potential risk of transmission of bat trypanosomes to domestic animals including, for example, dogs (known to manifest disease from *T. cruzi* and *T.* *evansi* infections) and livestock (which are severely affected by surra).

In the context of wildlife conservation, there is a significant knowledge gap regarding the clinical effects of bat trypanosomes in wildlife hosts in their natural environment. The well-known pathogens *T. cruzi* and *T. evansi* have the potential to cause disease in native mammals and a case report suggests *T. teixeirae* is pathogenic to bats. Determining any negative impacts of trypanosome species on wildlife conservation is challenging and may involve multiple aspects including parasite burden, immune competency and co-infections (or polyparasitism).

Indeed, the implications polyparasitism may have on a single vertebrate host, together with understanding multi-host parasite dynamics, are key research priorities in the 21st century. This is in addition to continuous baseline research on single host–parasite dynamics which is essential particularly for newly described species. Future epidemiological studies should adopt traditional multi-locus approaches associated with advanced molecular technologies such as amplicon NGS, to screen bats and candidate vectors for trypanosomes. In addition, for confirmation of the vectorial role of some arthropod species harbouring trypanosomes, experimental transmission studies are required. However, it is acknowledged that this is frequently both logistically and ethically challenging.

This review used a One Health approach to synthesise available information and explore in more detail the interesting and complex eco-epidemiology of the main bat trypanosome, *T. cruzi.* Overall, research shows that sylvatic and domestic transmission cycles of *T. cruzi* have been facilitated by human encroachment and consequent exchange of niches between bats, terrestrial mammals, and invertebrate vectors. These studies also suggest some level of correlation between ecological patterns and intra-specific genetic diversity of this parasite. Nevertheless, attempts to establish specific links between a given subpopulation of *T. cruzi* with vector ecology, reservoir hosts and clinical manifestation of disease have not yet resulted in conclusive associations, particularly in sylvatic settings. To achieve this goal, long-term studies comprising a representative number of *T. cruzi* isolates and mammalian species from different biomes and habitats are required.

In conclusion, current and future research and surveillance of bat trypanosomes, hosts, vectors and their shared environment will provide an opportunity for improved disease prevention and control, of these parasites by mitigation of biosecurity risks and potential spill-over events, ultimately ensuring the health of humans, domestic animals and wildlife. It is important to emphasise, however, that while bats may pose a risk to human and animal health, their numerous ecological benefits such as pollination and insect control far outweigh their zoonotic disease transmission potential. Therefore, a reductionist approach to eradicate bats and their parasites cannot be justified; instead, disease control should rely on vector control in domestic areas and measures to mitigate the human encroachment to bats’ natural habitats. Importantly, education strategies that take into consideration social and cultural characteristics of communities in endemic areas for bat pathogens, including *T. cruzi*, are essential to promote One Health.

## Figures and Tables

**Table 1:** *Trypanosoma* (*Schizotrypanum*) species/genotypes reported in Chiroptera. Geographical origins, Chiroptera host family, known infective host species, transmission routes and clinical significance provided.

*Trypanosoma (Schizotrypanum)* sp.	Geographic Distribution	Bats (Family)	Other Host Species	Transmission Routes	Clinical Significance	Ref.
**Bat-restricted trypanosomes**						
*T. cruzi marinkellei*	Central/South America	Phyllostomidae Vespertilionidae Mormoopidae	Unknown	Biological	Unknown	[30,48]
*T. erneyi*	Africa	Molossidae	Unknown	Unknown	Unknown	[49]
*T. livingstonei*	Africa	Rhinolophidae Hipposideridae	Unknown	Unknown	Unknown	[51]
*T. cf. livingstonei*	Africa	Nycteridae Miniopteridae	Unknown	Unknown	Unknown	[60]
*T. madeirae*	Central/South America	Phyllostomidae	Unknown	Unknown	Unknown	[69]
*T. teixeirae*	Australia	Pterpodidae	Unknown	Unknown	Pathogenic	[47]
*T. vespertillionis*	Europe	Vespertilionidae	Unknown	Biological	Unknown	[48]
*T. vespertilionis*-like G1	Africa	Vespertilionidae	Unknown	Unknown	Unknown	[26]
*T. vespertilionis*-like G2	Africa	Vespertilionidae	Unknown	Unknown	Unknown	[26]
*T. wauwau*	Central/South America	Mormoopidae	Unknown	Unknown	Unknown	[70]
*Trypanosoma sp.* HochG3	Africa	Vespertilionidae	Unknown	Unknown	Unknown	[26]
T. sp. bat	Africa	Pteropodidae	Unknown	Unknown	Unknown	[54]
*Trypanosoma sp.* NeoBat	Central/South America	Phyllostomidae	Unknown	Unknown	Unknown	[54]
*T. sp.* 1	Africa Europe	Miniopteridae	Unknown	Unknown	Unknown	[60]
*T. sp.* 2	Africa	Miniopteridae	Unknown	Unknown	Unknown	[60]
**Zoonotic bat trypanosomes**						
*T. cruzi cruzi* DTU (TcI–TcIV)	North/Central/ South America	Phyllostomidae Vespertilionidae Noctionidae Mormoopidae Thyropteridae	Generalist	Biological Mechanical Vertical	Pathogenic	[26,48]
*T. rangeli*	Central/South America	Phyllostomidae	Generalist	Biological Mechanical	Non-pathogenic	[48]
*T. dionisii*	Cosmopolitan	Molossidae Phyllostomidae Vespertilionidae	Human Opossum	Biological	Potentially Pathogenic	[16,18]
*T. sp.* TcBat	Central/South America	Pteropodidae	Human	Unknown	Non-pathogenic	[70]

**Table 2:** *Trypanosoma* species of Chiroptera outside the *Schizotrypanum* subgenus.

*Trypanosoma* sp.	Host Species	Geographic Location	Ref.
***Megatrypanum***			
*T. heybergi*	*Nycteris hispida* *Nycteris capensis* *Pipistrellus kuhlii*	Congo Kenya Egypt	[24,81]
*T. incertum*	*Pipistrellus pipistrellu*	United Kingdom	[32]
*T. leleupi*	*Hipposideros caffer*	Congo	[5,82]
*T. leonidasdeanei*	*Saccopterix bilineata*	Costa Rica	[5,83]
*T. lizae*	*Hipposideros cyclops*	Gabon	[32,84]
*T. magadermae*	*Lavia frons* *P. kuhlii*	Sudan Egypt	[24,85]
*T. megachiropterum*	*Pteropus tonganus*	Tonga	[32,86]
*T. morinorum*	*Asellia tridens*	Senegal	[5,87]
*T. mpapuense*	*Nycteris aethiopica*	Tanganyika	[5]
*T. pessoai*	*D. rotundus*	Brazil	[5,19]
*T. pifanoi*	*Artibeus lituratus Phillostomus hastatus*	Colombia	[5,88]
*T. possoai*	*Desmodus rotundus* *P. kuhlii*	Brazil	[19]
*T. rhinopoma*	*Rhinopoma hardwickei*	India	[32,89]
*T. scotophila*	*Scotophilus heathi horsfield*	China	[32,90]
*T. thomasi*	*Nycteris macrotis*	Congo	[91,92]
***Herpetosoma***			
*T. lineatum*	*Vampyrops lineatum*	Venezuela	[93]
*T. longiflagellum*	*Taphozous nudiventris*	Iraq	[93]
*T. anauwa*	*Miniopterus tristris*	Papua New Guinea	[27]
***Trypanozoon***			
*T. evansi*	*D. rotundus*	Latin America	[5]
**Uncharacterised subgenus**			
*T. vegrandis*	*Chalinolobus gouldii* *Nyctophilus geoffroyi* *Pteropus Alecto* *Pteropus scapulatus*	Australia	[45]

## Data Availability

Not applicable.

## References

[1] Wu Y.C., Chen C.S., Chan Y.J. (2020). The outbreak of COVID-19: An overview. J. Chin. Med. Assoc..

[2] Mann S., Frasca K., Scherrer S., Henao-Martinez A.F., Newman S., Ramanan P., Suarez J.A. (2021). A Review of Leishmaniasis: Current Knowledge and Future Directions. Curr. Trop. Med. Rep..

[3] Steverding D. (2014). The history of Chagas disease. Parasit. Vectors.

[4] Wang P.G., Kudelko M., Kwok K.T., Bruzzone R., Nal B. (2016). Molecular dissection of dengue virus egress: Involvement of the class II ARF small GTPase. Hong Kong Med. J..

[5] Hoare C.A. (1972). The Trypanosomes of Mammals: A Zoological Monograph.

[6] Aregawi W.G., Agga G.E., Abdi R.D., Buscher P. (2019). Systematic review and meta-analysis on the global distribution, host range, and prevalence of *Trypanosoma evansi*. Parasit. Vectors.

[7] Munoz J., Coll O., Juncosa T., Verges M., del Pino M., Fumado V., Bosch J., Posada E.J., Hernandez S., Fisa R. (2009). Prevalence and vertical transmission of *Trypanosoma cruzi* infection among pregnant Latin American women attending 2 maternity clinics in Barcelona, Spain. Clin. Infect. Dis..

[8] Nobrega A.A., Garcia M.H., Tatto E., Obara M.T., Costa E., Sobel J., Araujo W.N. (2009). Oral transmission of Chagas disease by consumption of acai palm fruit, Brazil. Emerg. Infect. Dis..

[9] Isaac C., Ciosi M., Hamilton A., Scullion K.M., Dede P., Igbinosa I.B., Nmorsi O.P., Masiga D., Turner C.M. (2016). Molecular identification of different trypanosome species and subspecies in tsetse flies of northern Nigeria. Parasit. Vectors.

[10] Reid S.A. (2002). *Trypanosoma evansi* control and containment in Australasia. Trends Parasitol..

[11] Sulkin S.E., Allen R. (1974). Virus infections in bats. Monogr. Virol..

[12] Turmelle A.S., Olival K.J. (2009). Correlates of viral richness in bats (order Chiroptera). Ecohealth.

[13] Calisher C.H., Childs J.E., Field H.E., Holmes K.V., Schountz T. (2006). Bats: Important reservoir hosts of emerging viruses. Clin. Microbiol. Rev..

[14] Hayman D.T., Bowen R.A., Cryan P.M., McCracken G.F., O’Shea T.J., Peel A.J., Gilbert A., Webb C.T., Wood J.L. (2013). Ecology of zoonotic infectious diseases in bats: Current knowledge and future directions. Zoonoses Public Health.

[15] Latinne A., Hu B., Olival K.J., Zhu G., Zhang L., Li H., Chmura A.A., Field H.E., Zambrana-Torrelio C., Epstein J.H. (2020). Origin and cross-species transmission of bat coronaviruses in China. Nat. Commun.

[16] Dario M.A., Lisboa C.V., Costa L.M., Moratelli R., Nascimento M.P., Costa L.P., Leite Y.L.R., Llewellyn M.S., Xavier S., Roque A.L.R. (2017). High *Trypanosoma* spp. diversity is maintained by bats and triatomines in Espirito Santo state, Brazil. PLoS ONE.

[17] Dario M.A., Pavan M.G., Rodrigues M.S., Lisboa C.V., Kluyber D., Desbiez A.L.J., Herrera H.M., Roque A.L.R., Lima L., Teixeira M.M.G. (2021). *Trypanosoma rangeli* Genetic, Mammalian Hosts, and Geographical Diversity from Five Brazilian Biomes. Pathogens.

[18] Dario M.A., Rodrigues M.S., Barros J.H.D., Xavier S.C.D., D’Andrea P.S., Roque A.L.R., Jansen A.M. (2016). Ecological scenario and *Trypanosoma cruzi* DTU characterization of a fatal acute Chagas disease case transmitted orally (Espirito Santo state, Brazil). Parasite Vector.

[19] Dean L.M., Sugay W. (1963). Trypanosoma possoai n. sp. In vampire bats Desmodus rotundus from the state of San Paulo, Brazil. Rev. Inst. Med. St. Paulo.

[20] Desquesnes M., Dargantes A., Lai D.H., Lun Z.R., Holzmuller P., Jittapalapong S. (2013). *Trypanosoma evansi* and surra: A review and perspectives on transmission, epidemiology and control, impact, and zoonotic aspects. Biomed. Res. Int..

[21] Dias J.C.P., Schofeild C.J. (2004). Control of Triatominae.

[22] Dos Santos F.C.B., Lisboa C.V., Xavier S.C.C., Dario M.A., Verde R.S., Calouro A.M., Roque A.L.R., Jansen A.M. (2018). *Trypanosoma* sp. diversity in Amazonian bats (Chiroptera; Mammalia) from Acre State, Brazil. Parasitology.

[23] Egan S.L., Taylor C.L., Austen J.M., Banks P.B., Ahlstrom L.A., Ryan U.M., Irwin P.J., Oskam C.L. (2020). Molecular identification of the *Trypanosoma* (*Herpetosoma*) lewisi clade in black rats (*Rattus rattus*) from Australia. Parasitol Res..

[24] El-Rahman A.R., Monib Mel S., Hassan A.A., Ghanam M.E., Shataat M.A., el-Damarany M. (2001). Studies on the *Megatrypanum* trypanosomes of the Egyptian bat (*Pipistrellum kuhl*i) from Sohag Governorate, Egypt. J. Egypt Soc. Parasitol..

[25] Ellis J., Barratt J., Kaufer A., Pearn L., Armstrong B., Johnson M., Park Y., Downey L., Cao M., Neill L. (2021). A new subspecies of *Trypanosoma cyclops* found in the Australian terrestrial leech *Chtonobdella bilineata*. Parasitology.

[26] Espinosa-Alvarez O., Ortiz P.A., Lima L., Costa-Martins A.G., Serrano M.G., Herder S., Buck G.A., Camargo E.P., Hamilton P.B., Stevens J.R. (2018). *Trypanosoma rangeli* is phylogenetically closer to Old World trypanosomes than to *Trypanosoma cruzi*. Int. J. Parasitol..

[27] Ewers W.H. (1974). *Trypanosoma aunawa* sp. n. from an insectivorous bat, *Miniopterus tristris*, in New Guinea, which may be transmitted by a leech. J. Parasitol..

[28] Eybpoosh S., Haghdoost A.A., Mostafavi E., Bahrampour A., Azadmanesh K., Zolala F. (2017). Molecular epidemiology of infectious diseases. Electron. Physician.

[29] Ferreira Lde L., Pereira M.H., Guarneri A.A. (2015). Revisiting *Trypanosoma rangeli* Transmission Involving Susceptible and Non-Susceptible Hosts. PLoS ONE.

[30] Franzen O., Talavera-Lopez C., Ochaya S., Butler C.E., Messenger L.A., Lewis M.D., Llewellyn M.S., Marinkelle C.J., Tyler K.M., Miles M.A. (2012). Comparative genomic analysis of human infective *Trypanosoma cruzi* lineages with the bat-restricted subspecies *T. cruzi marinkellei*. BMC Genom..

[31] Garcia E.S., Castro D.P., Figueiredo M.B., Azambuja P. (2012). Parasite-mediated interactions within the insect vector: *Trypanosoma rangeli* strategies. Parasit. Vectors.

[32] Gardner R.A., Molyneux D.H. (1988). *Trypanosoma* (*Megatrypanum*) *incertum* from *Pipistrellus pipistrellus*: Development and transmission by cimicid bugs. Parasitology.

[33] Gibson W., Bingle L., Blendeman W., Brown J., Wood J., Stevens J. (2000). Structure and sequence variation of the trypanosome spliced leader transcript. Mol. Biochem. Parasitol..

[34] Grisard E.C., Sturm N.R., Campbell D.A. (2003). A new species of trypanosome, *Trypanosoma desterrensis* sp. n., isolated from South American bats. Parasitology.

[35] Hamilton P.B., Cruickshank C., Stevens J.R., Teixeira M.M., Mathews F. (2012). Parasites reveal movement of bats between the New and Old Worlds. Mol. Phylogenet. Evol..

[36] Hamilton P.B., Gibson W.C., Stevens J.R. (2007). Patterns of co-evolution between trypanosomes and their hosts deduced from ribosomal RNA and protein-coding gene phylogenies. Mol. Phylogenet. Evol..

[37] Hamilton P.B., Stevens J.R., Telleria J., Tibayrenc M. (2017). 15—Classification and Phylogeny of Trypanosoma Cruzi. American Trypanosomiasis Chagas Disease.

[38] Hamilton P.B., Stevens J.R., Gaunt M.W., Gidley J., Gibson W.C. (2004). Trypanosomes are monophyletic: Evidence from genes for glyceraldehyde phosphate dehydrogenase and small subunit ribosomal RNA. Int. J. Parasitol..

[39] Hamilton P.B., Stevens J.R., Gidley J., Holz P., Gibson W.C. (2005). A new lineage of trypanosomes from Australian vertebrates and terrestrial bloodsucking leeches (Haemadipsidae). Int. J. Parasitol..

[40] Hamilton P.B., Teixeira M.M., Stevens J.R. (2012). The evolution of *Trypanosoma cruz*i: The ‘bat seeding’ hypothesis. Trends Parasitol..

[41] Hashimoto K., Schofeild C.J. (2012). Elimination of *Rhodnius prolixus* in Central America. Parasites Vectors.

[42] Hoare C.A. (1965). Vampire bats as vectors and hosts of equine and bovine trypanosomes. Acta Trop..

[43] Hodo C.L., Hamer S.A. (2017). Toward an Ecological Framework for Assessing Reservoirs of Vector-Borne Pathogens: Wildlife Reservoirs of *Trypanosoma cruzi* across the Southern United States. ILAR J..

[44] Jansen A.M., Xavier S., Roque A.L.R. (2018). *Trypanosoma cruzi* transmission in the wild and its most important reservoir hosts in Brazil. Parasit. Vectors.

[45] Austen J.M., O’Dea M., Jackson B., Ryan U. (2015). High prevalence of *Trypanosoma vegrandis* in bats from Western Australia. Vet. Parasitol..

[46] Austen J.M., Van Kampen E., Egan S.L., O’Dea M.A., Jackson B., Ryan U.M., Irwin P.J., Prada D. (2020). First report of *Trypanosoma dionisii* (Trypanosomatidae) identified in Australia. Parasitology.

[47] Barbosa A.D., Mackie J.T., Stenner R., Gillett A., Irwin P., Ryan U. (2016). *Trypanosoma teixeirae*: A new species belonging to the *T. cruzi* clade causing trypanosomosis in an Australian little red flying fox (*Pteropus scapulatus*). Vet. Parasitol..

[48] Beltz L.A. (2017). Kinetoplastids and bats. Bats and Human Health.

[49] Lima L., Silva F.M., Neves L., Attias M., Takata C.S., Campaner M., de Souza W., Hamilton P.B., Teixeira M.M. (2012). Evolutionary insights from bat trypanosomes: Morphological, developmental and phylogenetic evidence of a new species, *Trypanosoma* (*Schizotrypanum*) *erneyi* sp. nov., in African bats closely related to *Trypanosoma* (*Schizotrypanum*) *cruzi* and allied species. Protist.

[50] Woo P.T., Hawkins J.D. (1975). Trypanosomes and experimental trypanosomaisis in East African bats. Acta Trop..

[51] Lima L., Espinosa-Alvarez O., Hamilton P.B., Neves L., Takata C.S., Campaner M., Attias M., de Souza W., Camargo E.P., Teixeira M.M. (2013). *Trypanosoma livingstonei*: A new species from African bats supports the bat seeding hypothesis for the *Trypanosoma cruzi* clade. Parasit. Vectors.

[52] Molyneux D.H., Kreier J.P., Baker J.R. (1991). Trypanosomes of bats. Parasitic Protozoa.

[53] Cavazzana M., Marcili A., Lima L., da Silva F.M., Junqueira A.C., Veludo H.H., Viola L.B., Campaner M., Nunes V.L., Paiva F. (2010). Phylogeographical, ecological and biological patterns shown by nuclear (ssrRNA and gGAPDH) and mitochondrial (Cyt b) genes of trypanosomes of the subgenus *Schizotrypanum* parasitic in Brazilian bats. Int. J. Parasitol..

[54] Lima L., Espinosa-Alvarez O., Pinto C.M., Cavazzana M., Pavan A.C., Carranza J.C., Lim B.K., Campaner M., Takata C.S., Camargo E.P. (2015). New insights into the evolution of the *Trypanosoma cruzi* clade provided by a new trypanosome species tightly linked to Neotropical Pteronotus bats and related to an Australian lineage of trypanosomes. Parasit. Vectors.

[55] Villena F.E., Gomez-Puerta L.A., Jhonston E.J., Del Alcazar O.M., Maguina J.L., Albujar C., Laguna-Torres V.A., Recuenco S.E., Ballard S.B., Ampuero J.S. (2018). First Report of *Trypanosoma cruzi* Infection in Salivary Gland of Bats from the Peruvian Amazon. Am. J. Trop. Med. Hyg..

[56] Kostygov A.Y., Karnkowska A., Votypka J., Tashyreva D., Maciszewski K., Yurchenko V., Lukes J. (2021). Euglenozoa: Taxonomy, diversity and ecology, symbioses and viruses. Open Biol..

[57] Mafie E., Rupa F.H., Takano A., Suzuki K., Maeda K., Sato H. (2018). First record of *Trypanosoma dionisii* of the *T. cruzi* clade from the Eastern bent-winged bat (*Miniopterus fuliginosus*) in the Far East. Parasitol. Res..

[58] Stevens J.R., Teixeira M.M., Bingle L.E., Gibson W.C. (1999). The taxonomic position and evolutionary relationships of *Trypanosoma rangeli*. Int. J. Parasitol..

[59] Wang L.J., Han H.J., Zhao M., Liu J.W., Luo L.M., Wen H.L., Qin X.R., Zhou C.M., Qi R., Yu H. (2019). *Trypanosoma dionisii* in insectivorous bats from northern China. Acta Trop..

[60] Clement L., Dietrich M., Markotter W., Fasel N.J., Monadjem A., Lopez-Baucells A., Scaravelli D., Theou P., Pigeault R., Ruedi M. (2020). Out of Africa: The origins of the protozoan blood parasites of the *Trypanosoma cruzi* clade found in bats from Africa. Mol. Phylogenet. Evol..

[61] Cottontail V.M., Kalko E.K., Cottontail I., Wellinghausen N., Tschapka M., Perkins S.L., Pinto C.M. (2014). High local diversity of *Trypanosoma* in a common bat species, and implications for the biogeography and taxonomy of the *T. cruzi* clade. PLoS ONE.

[62] Stevens J.R., Gibson W. (1999). The molecular evolution of trypanosomes. Parasitol. Today.

[63] Da Silva F.M., Noyes H., Campaner M., Junqueira A.C., Coura J.R., Anez N., Shaw J.J., Stevens J.R., Teixeira M.M. (2004). Phylogeny, taxonomy and grouping of *Trypanosoma rangeli* isolates from man, triatomines and sylvatic mammals from widespread geographical origin based on SSU and ITS ribosomal sequences. Parasitology.

[64] Zingales B., Miles M.A., Campbell D.A., Tibayrenc M., Macedo A.M., Teixeira M.M., Schijman A.G., Llewellyn M.S., Lages-Silva E., Machado C.R. (2012). The revised *Trypanosoma cruzi* subspecific nomenclature: Rationale, epidemiological relevance and research applications. Infect. Genet. Evol..

[65] Baker J.R., Miles M.A., Godfrey D.G., Barrett T.V. (1978). Biochemical characterization of some species of *Trypanosoma* (*Schizotrypanum*) from Bats (Microchiroptera). Am. J. Trop. Med. Hyg..

[66] Barbosa A., Austen J., Gillett A., Warren K., Paparini A., Irwin P., Ryan U. (2016). First report of *Trypanosoma vegrandis* in koalas (*Phascolarctos cinereus*). Parasitol. Int..

[67] Bradwell K.R., Koparde V.N., Matveyev A.V., Serrano M.G., Alves J.M.P., Parikh H., Huang B., Lee V., Espinosa-Alvarez O., Ortiz P.A. (2018). Genomic comparison of *Trypanosoma conorhini* and *Trypanosoma rangeli* to *Trypanosoma cruzi* strains of high and low virulence. BMC Genom..

[68] Botero A., Cooper C., Thompson C.K., Clode P.L., Rose K., Thompson R.C. (2016). Morphological and Phylogenetic Description of *Trypanosoma noyesi* sp. nov.: An Australian Wildlife Trypanosome within the *T. cruzi* Clade. Protist.

[69] Barros J.H.S., Lima L., Schubach A.O., Teixeira M.M.G. (2019). *Trypanosoma madeirae* sp. n.: A species of the clade *T. cruzi* associated with the neotropical common vampire bat *Desmodus rotundus*. Int. J. Parasitol. Parasites Wildl..

[70] Ramirez J.D., Tapia-Calle G., Munoz-Cruz G., Poveda C., Rendon L.M., Hincapie E., Guhl F. (2014). Trypanosome species in neo-tropical bats: Biological, evolutionary and epidemiological implications. Infect. Genet. Evol..

[71] Stevens J.R., Noyes H.A., Schofield C.J., Gibson W. (2001). The molecular evolution of Trypanosomatidae. Adv. Parasitol..

[72] Maslov D.A., Opperdoes F.R., Kostygov A.Y., Hashimi H., Lukes J., Yurchenko V. (2019). Recent advances in trypanosomatid research: Genome organization, expression, metabolism, taxonomy and evolution. Parasitology.

[73] Votypka J., d’Avila-Levy C.M., Grellier P., Maslov D.A., Lukes J., Yurchenko V. (2015). New approaches to systematics of Trypanosomatidae: Criteria for taxonomic (re)description. Trends Parasitol..

[74] Thompson C.K., Botero A., Wayne A.F., Godfrey S.S., Lymbery A.J., Thompson R.C. (2013). Morphological polymorphism of *Trypanosoma copemani* and description of the genetically diverse *T. vegrandis* sp. nov. from the critically endangered Australian potoroid, the brush-tailed bettong (*Bettongia penicillata* (Gray, 1837)). Parasit. Vectors.

[75] Austen J.M., Jefferies R., Friend J.A., Ryan U., Adams P., Reid S.A. (2009). Morphological and molecular characterization of *Trypanosoma copemani* n. sp. (Trypanosomatidae) isolated from Gilbert’s potoroo (*Potorous gilbertii*) and quokka (*Setonix brachyurus*). Parasitology.

[76] McInnes L.M., Hanger J., Simmons G., Reid S.A., Ryan U.M. (2011). Novel trypanosome *Trypanosoma gilletti* sp. (Euglenozoa: Trypanosomatidae) and the extension of the host range of *Trypanosoma copemani* to include the koala (*Phascolarctos cinereus*). Parasitology.

[77] Breinl A. (1913). Parasite protozoa encountered in the blood of Australian native animals. Aust. Inst. Trop. Med..

[78] Mackerras M.J. (1959). The haematozoa of Australian mammals. Aust. J. Zool..

[79] Bower S.M., Woo P.T.K. (1981). Development of *Trypanosoma* (*Schizotrypanum*) *hedricki* in *Cimex brevis* (Hemiptera: Cimicidae). Can. J. Zool..

[80] Paterson W.B., Woo P.T.K. (1983). An ultrastructural study of the culture forms of *Trypanosoma* (*Schizotrypanum*) *myoti*. Can. J. Zool..

[81] Rodhain J. (1923). Trypanosome d’un chéiroptère insectivore *Nycteris hispida* Schreber au Congo Beige. Bull. Soc. Pathol. Exot..

[82] Rodhain J. (1951). *Trypanosoma leleupi* n. sp. parasite de *Hipposideros caffer* au Katanga. Annls. Parasit. Hum. Comp..

[83] Zeledon R., Rosabal R. (1969). *Trypanosoma leonidasdeanei* sp. n. in insectivorous bats of Costa Rica. Ann. Trop. Med. Parasit.

[84] Miltgen F., Landau I. (1979). *Trypanosoma* (*Megatrypanum*) *lizae* n.sp. un trypanosome ayant des formes geantes chez les microchiropteres *Hipposideros cyclops* au Gabo. Ann. Parasitolo Gie Hum. Comp..

[85] Wenyon C.M. (1909). Report of a Travelling Pathologist and Protozoologist.

[86] Marinkelle C.J. (1979). *Trypanosoma* (*Megatrypanum*) megachiropterum sp.n. from the flying fox. Pteropus tonganus Quoy and Galmard. J. Protozool..

[87] Leger M., Baury A. (1923). Trypanosome de la chauve-souris du *Senegal Hipposideros* tridens, Et. Geoff C.r.Skanc. Soc. Biol..

[88] Marinkelle C.J., Duarte C.A. (1968). *Trypanosoma pifanoi* n. sp. from Colombian bats. J. Protozool..

[89] Bandyopadhyay S., Ray R., Dasgupta B. (1982). A new species of *Trypanosoma* from an Indian insectivotous bat, *Rhinopoma hardwickei* Gray. Acta Protozool..

[90] Liao G.Y. (1982). Biology of a new trypanosome: Trypanosoma (Megatrypanum) scotophila sp.nov. from the bat Scotophilus heathi Horsfield. Malaria and Other Protozoal Infections.

[91] Keymer I.F. (1971). Blood Protozoa of Insectivores, Bats and Primates in Central Africa. J. Zool..

[92] Lips M., Rodhain J. (1956). Quelques hematozoaires de petits mammiferes du Haut-Katang. Annls. Parasit. Hum. Comp..

[93] Marinkelle C.J. (1977). *Trypanosoma (Herpetosoma) longiflagellum* sp.n. from the tomb bat, *Taphozous nudiventris*, from Iraq. J. Wildl. Dis..

[94] Austen J.M., Ryan U.M., Friend J.A., Ditcham W.G., Reid S.A. (2011). Vector of *Trypanosoma copemani* identified as *Ixodes* sp.. Parasitology.

[95] Latif A.A., Bakheit M.A., Mohamed A.E., Zweygarth E. (2004). High infection rates of the tick *Hyalomma anatolicum anatolicum* with *Trypanosoma theileri*. Onderstepoort J. Vet. Res..

[96] Luu L., Bown K.J., Palomar A.M., Kazimirova M., Bell-Sakyi L. (2020). Isolation and partial characterisation of a novel *Trypanosoma* from the tick *Ixodes ricinus*. Ticks Tick Borne Dis..

[97] Marotta R.C., Dos Santos P.N., Cordeiro M.D., Matos P.C.M., Barros J.H.D.S., Madeira M.F., Bell Sakyi L., Fonseca A.H. (2018). *Trypanosoma rhipicephalis* sp. nov. (Protozoa: Kinetoplastida) isolated from *Rhipicephalus microplus* (Acari: Ixodidae) ticks in Rio de Janeiro, Brazil. Parasitol. Open.

[98] Thekisoe O.M., Honda T., Fujita H., Battsetseg B., Hatta T., Fujisaki K., Sugimoto C., Inoue N. (2007). A trypanosome species isolated from naturally infected *Haemaphysalis hystricis* ticks in Kagoshima Prefecture, Japan. Parasitology.

[99] Szentiványi T., Christe P., Glaizot O. (2019). Bat flies and their microparasites:current knowledge and distribution. Front. Vet. Sci..

[100] Buscaglia C.A., Di Noia J.M. (2003). *Trypanosoma cruzi* clonal diversity and the epidemiology of Chagas’ disease. Microbes Infect..

[101] Marinkelle C.J. (1982). Developmental stages of *Trypanosoma cruzi*-like flagellates in *Cavernicola pilosa*. Rev. Biol. Trop..

[102] D’Alessandro A., Saravia N.G., Kreier J.P., Bake J.R. (1992). Trypanosoma rangeli. Parasitic Protozoa.

[103] Stevens J., Noyes H., Gibson W. (1998). The evolution of trypanosomes infecting humans and primates. Mem. Inst. Oswaldo Cruz.

[104] Salazar R., Castillo-Neyra R., Tustin A.W., Borrini-Mayori K., Naquira C., Levy M.Z. (2015). Bed bugs (*Cimex lectularius*) as vectors of *Trypanosoma cruzi*. Am. J. Trop. Med. Hyg..

[105] Van Den Berghe L., Chardome M., Peel E. (1963). An African bat trypanosome in *Stricticimex brevispinosus* Usinger, 1959. J. Protozool..

[106] Rodhain J. (1939). Mode de transmission de *Trypanosoma vespertilionis* Battaglia par les arthropodes. C. R. Seances Soc. Biol. Fil..

[107] Mcconnell E., Correa M. (1964). Trypanosomes + Other Microorganisms from Panamanian Phlebotomus Sandflies. J. Parasitol..

[108] Pereira K.S., Sciimidt F.L., Guaraldo A.M.A., Franco R.M.B., Dias V.L., Passos L.A.C. (2009). Chagas’ Disease as a Foodborne Illness. J. Food Prot..

[109] Oliveira M.P., Cortez M., Maeda F.Y., Fernandes M.C., Haapalainen E.F., Yoshida N., Mortara R.A. (2009). Unique behavior of *Trypanosoma dionisii* interacting with mammalian cells: Invasion, intracellular growth, and nuclear localization. Acta Trop..

[110] Caradonna K.L., Burleigh B.A. (2011). Mechanisms of host cell invasion by *Trypanosoma cruzi*. Adv. Parasitol..

[111] Machado F.S., Dutra W.O., Esper L., Gollob K.J., Teixeira M.M., Factor S.M., Weiss L.M., Nagajyothi F., Tanowitz H.B., Garg N.J. (2012). Current understanding of immunity to *Trypanosoma cruzi* infection and pathogenesis of Chagas disease. Semin. Immunopathol..

[112] Bonney K.M., Engman D.M. (2008). Chagas heart disease pathogenesis: One mechanism or many?. Curr. Mol. Med..

[113] Lukes J., Butenko A., Hashimi H., Maslov D.A., Votypka J., Yurchenko V. (2018). Trypanosomatids Are Much More than Just Trypanosomes: Clues from the Expanded Family Tree. Trends Parasitol..

[114] Marcondes M.C., Borelli P., Yoshida N., Russo M. (2000). Acute *Trypanosoma cruzi* infection is associated with anemia, thrombocytopenia, leukopenia, and bone marrow hypoplasia: Reversal by nifurtimox treatment. Microbes Infect..

[115] Murray M., Dexter T.M. (1988). Anaemia in bovine African trypanosomiasis. A review. Acta Trop..

[116] Noyes H.A., Alimohammadian M.H., Agaba M., Brass A., Fuchs H., Gailus-Durner V., Hulme H., Iraqi F., Kemp S., Rathkolb B. (2009). Mechanisms controlling anaemia in *Trypanosoma congolense* infected mice. PLoS ONE.

[117] Macedo A.M., Pena S.D. (1998). Genetic Variability of *Trypanosoma cruzi*:Implications for the Pathogenesis of Chagas Disease. Parasitol. Today.

[118] Perez C.J., Lymbery A.J., Thompson R.C. (2014). Chagas disease: The challenge of polyparasitism?. Trends Parasitol..

[119] Wibbelt G., Moore M.S., Schountz T., Voigt C.C. (2010). Emerging diseases in Chiroptera: Why bats?. Biol. Lett..

[120] Mackie J.T., Stenner R., Gillett A.K., Barbosa A., Ryan U., Irwin P.J. (2017). Trypanosomiasis in an Australian little red flying fox (*Pteropus scapulatus*). Aust. Vet. J..

[121] Garcia L., Ortiz S., Osorio G., Torrico M.C., Torrico F., Solari A. (2012). Phylogenetic analysis of Bolivian bat trypanosomes of the subgenus *Schizotrypanum* based on cytochrome B sequence and minicircle analyses. PLoS ONE.

[122] Lewis M.D., Llewellyn M.S., Yeo M., Acosta N., Gaunt M.W., Miles M.A. (2011). Recent, independent and anthropogenic origins of *Trypanosoma cruzi* hybrids. PLoS Negl. Trop. Dis..

[123] Calvo-Mendez M.L., Nogueda-Torres B., Alejandre-Aguilar R., Cortes-Jimenez M. (1994). Experimental *Trypanosoma cruzi* infection via contaminated water and food. Rev. Lat. Microbiol..

[124] Jansen A.M., Moriearty P.L., Castro B.G., Deane M.P. (1985). *Trypanosoma cruzi* in the opossum *Didelphis marsupialis*: An indirect fluorescent antibody test for the diagnosis and follow-up of natural and experimental infections. Trans. R Soc. Trop. Med. Hyg..

[125] Silva N.N., Clausell D.T., Nolibos H., Mello A.L., O’ssanal J., Rapone T., Snell T. (1968). Surto epidêmico de doença de Chagas com provável contaminação oral. Rev. Inst. Med. Trop Sao Paulo.

[126] Shikanai-Yasuda M.A., Carvalho N.B. (2012). Oral transmission of Chagas disease. Clin. Infect. Dis..

[127] Rodriguez-Morales A.J. (2008). Chagas disease: An emerging food-borne entity?. J. Infect. Dev. Countries.

[128] Pinto C.M., Kalko E.K., Cottontail I., Wellinghausen N., Cottontail V.M. (2012). TcBat a bat-exclusive lineage of *Trypanosoma cruzi* in the Panama Canal Zone, with comments on its classification and the use of the 18S rRNA gene for lineage identification. Infect. Genet. Evol..

[129] Carter G., Leffer L. (2015). Social Grooming in Bats: Are Vampire Bats Exceptional?. PLoS ONE.

[130] Caraballo A.J. (1996). Outbreak of vampire bat biting in a Venezuelan village. Rev. Saude Publica.

[131] Bergner L.M., Orton R.J., Benavides J.A., Becker D.J., Tello C., Biek R., Streicker D.G. (2020). Demographic and environmental drivers of metagenomic viral diversity in vampire bats. Mol. Ecol..

[132] Johnson P.D. (1933). A case of infection by Trypanosoma lewisi in a child. Trans. R Soc. Trop. Med. Hyg..

[133] Lun Z.R., Reid S.A., Lai D.H., Li F.J. (2009). Atypical human trypanosomiasis: A neglected disease or just an unlucky accident?. Trends Parasitol..

[134] Sarataphan N., Vongpakorn M., Nuansrichay B., Autarkool N., Keowkarnkah T., Rodtian P., Stich R.W., Jittapalapong S. (2007). Diagnosis of a *Trypanosoma lewis*i-like (*Herpetosoma*) infection in a sick infant from Thailand. J. Med. Microbiol..

[135] Joshi P.P., Shegokar V.R., Powar R.M., Herder S., Katti R., Salkar H.R., Dani V.S., Bhargava A., Jannin J., Truc P. (2005). Human trypanosomiasis caused by *Trypanosoma evansi* in India: The first case report. Am. J. Trop. Med. Hyg..

[136] Austen J.M., Reid S.A., Robinson D.R., Friend J.A., Ditcham W.G., Irwin P.J., Ryan U. (2015). Investigation of the morphological diversity of the potentially zoonotic *Trypanosoma copemani* in quokkas and Gilbert’s potoroos. Parasitology.

[137] Barbosa A.D., Austen J., Portas T.J., Friend J.A., Ahlstrom L.A., Oskam C.L., Ryan U.M., Irwin P.J. (2019). Sequence analyses at mitochondrial and nuclear loci reveal a novel Theileria sp. and aid in the phylogenetic resolution of piroplasms from Australian marsupials and ticks. PLoS ONE.

[138] Austen J.M., Paparini A., Reid S.A., Friend J.A., Ditcham W.G., Ryan U. (2016). Molecular characterization of native Australian trypanosomes in quokka (*Setonix brachyurus*) populations from Western Australia. Parasitol. Int..

[139] Ledezma A.P., Blandon R., Schijman A.G., Benatar A., Saldana A., Osuna A. (2020). Mixed infections by different *Trypanosoma cruzi* discrete typing units among Chagas disease patients in an endemic community in Panama. PLoS ONE.

[140] Burgos J.M., Altcheh J., Bisio M., Duffy T., Valadares H.M., Seidenstein M.E., Piccinali R., Freitas J.M., Levin M.J., Macchi L. (2007). Direct molecular profiling of minicircle signatures and lineages of *Trypanosoma cruzi* bloodstream populations causing congenital Chagas disease. Int. J. Parasitol..

[141] Fernandes O., Santos S.S., Cupolillo E., Mendonca B., Derre R., Junqueira A.C., Santos L.C., Sturm N.R., Naiff R.D., Barret T.V. (2001). A mini-exon multiplex polymerase chain reaction to distinguish the major groups of *Trypanosoma cruzi* and *T. rangeli* in the Brazilian Amazon. Trans. R Soc. Trop. Med. Hyg..

[142] Fernandes O., Sturm N.R., Derre R., Campbell D.A. (1998). The mini-exon gene: A genetic marker for zymodeme III of *Trypanosoma cruzi*. Mol. Biochem. Parasitol..

[143] Jaramillo N., Moreno J., Triana O., Arcos-Burgos M., Munoz S., Solari A. (1999). Genetic structure and phylogenetic relationships of Colombian *Trypanosoma cruzi* populations as determined by schizodeme and isoenzyme markers. Am. J. Trop. Med. Hyg..

[144] Triana O., Ortiz S., Dujardin J.C., Solari A. (2006). *Trypanosoma cruzi*: Variability of stocks from Colombia determined by molecular karyotype and minicircle Southern blot analysis. Exp. Parasitol..

[145] Rodrigues M.S., Lima L., Xavier S., Herrera H.M., Rocha F.L., Roque A.L.R., Teixeira M.M.G., Jansen A.M. (2019). Uncovering *Trypanosoma* spp. diversity of wild mammals by the use of DNA from blood clots. Int. J. Parasitol. Parasites Wildl..

[146] Duarte L.F., Florez O., Rincon G., Gonzalez C.I. (2014). Comparison of seven diagnostic tests to detect *Trypanosoma cruzi* infection in patients in chronic phase of Chagas disease. Colomb. Med. (Cali).

[147] Lewis M.D., Ma J., Yeo M., Carrasco H.J., Llewellyn M.S., Miles M.A. (2009). Genotyping of *Trypanosoma cruz*i: Systematic selection of assays allowing rapid and accurate discrimination of all known lineages. Am. J. Trop. Med. Hyg..

[148] Araujo C.A., Cabello P.H., Jansen A.M. (2007). Growth behaviour of two *Trypanosoma cruzi* strains in single and mixed infections: In vitro and in the intestinal tract of the blood-sucking bug, *Triatoma brasiliensis*. Acta Trop..

[149] Barbosa A.D., Gofton A.W., Paparini A., Codello A., Greay T., Gillett A., Warren K., Irwin P., Ryan U. (2017). Increased genetic diversity and prevalence of co-infection with *Trypanosoma* spp. in koalas (*Phascolarctos cinereus*) and their ticks identified using next-generation sequencing (NGS). PLoS ONE.

[150] Northover A.S., Godfrey S.S., Keatley S., Lymbery A.J., Wayne A.F., Cooper C., Pallant L., Morris K., Thompson R.C.A. (2019). Increased *Trypanosoma* spp. richness and prevalence of haemoparasite co-infection following translocation. Parasit. Vectors.

[151] Petney T.N., Andrews R.H. (1998). Multiparasite communities in animals and humans: Frequency, structure and pathogenic significance. Int. J. Parasitol..

[152] Pullan R., Brooker S. (2008). The health impact of polyparasitism in humans: Are we under-estimating the burden of parasitic diseases?. Parasitology.

[153] Grybchuk-Ieremenko A., Losev A., Kostygov A.Y., Lukes J., Yurchenko V. (2014). High prevalence of trypanosome co-infections in freshwater fishes. Folia Parasitol..

[154] Spodareva V.V., Grybchuk-Ieremenko A., Losev A., Votypka J., Lukes J., Yurchenko V., Kostygov A.Y. (2018). Diversity and evolution of anuran trypanosomes: Insights from the study o European species. Parasite. Vector.

[155] Yurchenko V.Y., Lukes J., Jirku M., Maslov D.A. (2009). Selective recovery of the cultivation-prone components from mixed trypanosomatid infections: A case of several novel species isolated from Neotropical Heteroptera. Int. J. Syst. Evol. Microbiol..

[156] Cooper C., Keatley S., Northover A., Gofton A.W., Brigg F., Lymbery A.J., Pallant L., Clode P.L., Thompson R.C.A. (2018). Next generation sequencing reveals widespread trypanosome diversity and polyparasitism in marsupials from Western Australia. Int. J. Parasitol. Parasites Wildl..

[157] Dario M.A., Moratelli R., Schwabl P., Jansen A.M., Llewellyn M.S. (2017). Small subunit ribosomal metabarcoding reveals extraordinary trypanosomatid diversity in Brazilian bats. PLoS Negl. Trop. Dis..

[158] Anez N., Crisante G., Soriano P.J. (2009). *Trypanosoma cruzi* congenital transmission in wild bats. Acta Trop..

[159] Noireau F., Diosque P., Jansen A.M. (2009). *Trypanosoma cruz*i: Adaptation to its vectors and its hosts. Vet. Res..

[160] Balouz V., Melli L.J., Volcovich R., Moscatelli G., Moroni S., Gonzalez N., Ballering G., Bisio M., Ciocchini A.E., Buscaglia C.A. (2017). The Trypomastigote Small Surface Antigen from *Trypanosoma cruzi* Improves Treatment Evaluation and Diagnosis in Pediatric Chagas Disease. J. Clin. Microbiol..

[161] Eisenstein M. (2016). Disease: Poverty and pathogens. Nature.

[162] Lidani K.C.F., Andrade F.A., Bavia L., Damasceno F.S., Beltrame M.H., Messias-Reason I.J., Sandri T.L. (2019). Chagas Disease: From Discovery to a Worldwide Health Problem. Front. Public Health.

[163] Lisboa C.V., Pinho A.P., Herrera H.M., Gerhardt M., Cupolillo E., Jansen A.M. (2008). *Trypanosoma cruzi* (Kinetoplastida, Trypanosomatidae) genotypes in neotropical bats in Brazil. Vet. Parasitol..

[164] Jansen A.M., Santos de Pinho A.P., Lisboa C.V., Cupolillo E., Mangia R.H., Fernandes O. (1999). The sylvatic cycle of *Trypanosoma cruzi*: A still unsolved puzzle. Mem. Inst. Oswaldo Cruz.

[165] Samudio F., Ortega-Barria E., Saldana A., Calzada J. (2007). Predominance of *Trypanosoma cruz*i I among Panamanian sylvatic isolates. Acta Trop..

[166] Roellig D.M., Brown E.L., Barnabe C., Tibayrenc M., Steurer F.J., Yabsley M.J. (2008). Molecular typing of *Trypanosoma cruzi* isolates, United States. Emerg. Infect. Dis..

[167] Bosseno M.F., Barnabe C., Magallon Gastelum E., Lozano Kasten F., Ramsey J., Espinoza B., Breniere S.F. (2002). Predominance of *Trypanosoma cruzi* lineage I in Mexico. J. Clin. Microbiol..

[168] Anez N., Crisante G., da Silva F.M., Rojas A., Carrasco H., Umezawa E.S., Stolf A.M., Ramirez J.L., Teixeira M.M. (2004). Predominance of lineage I among *Trypanosoma cruzi* isolates from Venezuelan patients with different clinical profiles of acute Chagas’ disease. Trop. Med. Int. Health.

[169] Cuervo P., Cupolillo E., Segura I., Saravia N., Fernandes O. (2002). Genetic diversity of Colombian sylvatic *Trypanosoma cruzi* isolates revealed by the ribosomal DNA. Mem. Inst. Oswaldo Cruz.

[170] Herrera C., Bargues M.D., Fajardo A., Montilla M., Triana O., Vallejo G.A., Guhl F. (2007). Identifying four *Trypanosoma cruzi* I isolate haplotypes from different geographic regions in Colombia. Infect. Genet. Evol..

[171] Miles M.A., Cedillos R.A., Povoa M.M., de Souza A.A., Prata A., Macedo V. (1981). Do radically dissimilar *Trypanosoma cruzi* strains (zymodemes) cause Venezuelan and Brazilian forms of Chagas’ disease?. Lancet.

[172] Marcili A., Lima L., Cavazzana M., Junqueira A.C., Veludo H.H., Maia Da Silva F., Campaner M., Paiva F., Nunes V.L., Teixeira M.M. (2009). A new genotype of *Trypanosoma cruzi* associated with bats evidenced by phylogenetic analyses using SSU rDNA, cytochrome b and Histone H2B genes and genotyping based on ITS1 rDNA. Parasitology.

[173] Yeo M., Acosta N., Llewellyn M., Sanchez H., Adamson S., Miles G.A., Lopez E., Gonzalez N., Patterson J.S., Gaunt M.W. (2005). Origins of Chagas disease: Didelphis species are natural hosts of *Trypanosoma cruzi* I and armadillos hosts of Trypanosoma cruzi II, including hybrids. Int. J. Parasitol..

[174] Guhl F., Auderheide A., Ramirez J.D. (2014). From ancient to contemporary molecular eco-epidemiology of Chagas disease in the Americas. Int. J. Parasitol..

[175] Ramirez J.D., Hernandez C., Montilla M., Zambrano P., Florez A.C., Parra E., Cucunuba Z.M. (2014). First report of human *Trypanosoma cruzi* infection attributed to TcBat genotype. Zoonoses Public Health.

[176] Coura J.R., Junqueira A.C. (2015). Ecological diversity of *Trypanosoma cruzi* transmission in the Amazon basin. The main scenaries in the Brazilian Amazon. Acta Trop..

[177] Lent H., Wygodzinsky W. (1979). Revision of the Triatominae (Hemiptera, Reduviidae), and their significance as vectors of Chagas’ disease. Bull. AMNH.

[178] Gaunt M., Miles M. (2000). The ecotopes and evolution of triatomine bugs (Triatominae) and their associated trypanosomes. Mem. Inst. Oswaldo Cruz.

[179] Miles M.A., Llewellyn M.S., Lewis M.D., Yeo M., Baleela R., Fitzpatrick S., Gaunt M.W., Mauricio I.L. (2009). The molecular epidemiology and phylogeography of *Trypanosoma cruzi* and parallel research on *Leishmania*: Looking back and to the future. Parasitology.

[180] Gurtler R.E., Cardinal M.V. (2015). Reservoir host competence and the role of domestic and commensal hosts in the transmission of *Trypanosoma cruzi*. Acta Trop..

[181] Mejia-Jaramillo A.M., Agudelo-Uribe L.A., Dib J.C., Ortiz S., Solari A., Triana-Chavez O. (2014). Genotyping of *Trypanosoma cruzi* in a hyper-endemic area of Colombia reveals an overlap among domestic and sylvatic cycles of Chagas disease. Parasit. Vectors.

[182] Guerenstein P.G., Lazzari C.R. (2009). Host-seeking: How triatomines acquire and make use of information to find blood. Acta Trop..

[183] Arias-Giraldo L.M., Munoz M., Hernandez C., Herrera G., Velasquez-Ortiz N., Cantillo-Barraza O., Urbano P., Cuervo A., Ramirez J.D. (2020). Identification of blood-feeding sources in *Panstrongylus*, *Psammolestes*, *Rhodnius* and *Triatoma* using amplicon-based next-generation sequencing. Parasit. Vectors.

[184] Thomas M.E., Rasweiler Iv J.J., D’Alessandro A. (2007). Experimental transmission of the parasitic flagellates *Trypanosoma cruzi* and *Trypanosoma rangeli* between triatomine bugs or mice and captive neotropical bats. Mem. Inst. Oswaldo Cruz.

[185] Thompson C.K., Thompson R.C.A. (2015). Trypanosomes of Australian Mammals: Knowledge Gaps Regarding Transmission and Biosecurity. Trends Parasitol..

[186] Thompson R.C.A. (2018). Exotic Parasite Threats to Australia’s Biosecurity-Trade, Health, and Conservation. Trop. Med. Infect. Dis..

[187] Noyes H.A., Stevens J.R., Teixeira M., Phelan J., Holz P. (1999). A nested PCR for the ssrRNA gene detects *Trypanosoma binneyi* in the platypus and *Trypanosoma* sp. in wombats and kangaroos in Australia. Int. J. Parasitol..

[188] Backhouse T.C., Bolliger A. (1951). Transmission of Chagas’ disease to the Australian marsupial *Trichosurus Vulpecula*. Trans. R Soc. Trop. Med. Hyg..

